# Cancer treatment by radioimmunotherapy: insights from a dynamical model of cancer stem cells and hypoxia effects

**DOI:** 10.1038/s41598-026-47796-w

**Published:** 2026-04-13

**Authors:** Alain Mvogo, Frank Eric Essongo, Germain Hubert Ben-Bolie

**Affiliations:** 1 Laboratory of Biophysics, Department of Physics, Faculty of Science, University of Yaounde I, P.O. Box 812, Yaounde, Cameroon; 2 Laboratory of Nuclear Physics, Dosimetry and Radiation Protection, Department of Physics, Faculty of Science, University of Yaounde I, P.O. Box 812, Yaounde, Cameroon

**Keywords:** Cancer model, Radioimmunotherapy, Biological effective clearance half-life, Tumor control probability, Hypoxia, Circular patterns, Cancer, Oncology

## Abstract

Cancer remains a major challenge for conventional treatments. This is due to the resistance mechanisms driven by cancer stem cells (CSCs) which sustain tumor growth. In this work, we investigate both analytically and computationally the effects of radioimmunotherapy (RIT), a cutting-edge technique that uses radiolabeled antibodies to precisely target and irradiate cancer cells. The work considers time delay modeling and the interactions between microRNAs and differentiated cancer cells (DCs). We evaluate the effects of extrapolated dose rates from four important radionuclides including yttrium-90 ($^{90}\textrm{Y}$), lutetium-177 ($^{177}\textrm{Lu}$), iodine-131 ($^{131}\textrm{I}$) and actinium-225 ($^{225}\textrm{Ac}$) in the preventive treatment of cancer before recurrence. A sensitivity analysis of model parameters is also performed to assess the robustness of the predictions and to identify the most influential biological and physical variables. Using the linear-quadratic formalism, we compare their biological effective dose, surviving fraction, and tumor control probability. The results demonstrate that an extrapolated initial dose of 165 $\mathrm {Gy.year^{-1}}$ leads to an eradication of CSCs using $^{225}\textrm{Ac}$ and $^{177}\textrm{Lu}$ within 1.4636 year and 1.5736 year, respectively. Similarly, DCs are eliminated with $^{225}\textrm{Ac}$ and $^{177}\textrm{Lu}$ over treatment durations of 0.9396 year and 1.0496 year, respectively. These results highlight the potent effects of $^{225}\textrm{Ac}$ and $^{177}\textrm{Lu}$ in targeting CSCs and DCs at this dose rate. Under these conditions, microRNAs act as tumor suppressors, thus preventing pro-tumorigenic effects. Exceeding the dose threshold (beyond 165 $\mathrm {Gy.year^{-1}}$) disrupts the therapeutic balance with an efficacy which decreases progressively. For the doses above 326 $\mathrm {Gy.year^{-1}}$, the overproliferation of CSCs and DCs is observed with an oncogenic behavior of microRNAs. We further examine the role of tumor oxygenation in modulating RIT efficacy. The results reveal that enhancing oxygen availability significantly increases CSC radiosensitivity, which is otherwise reduced under hypoxic conditions. The results of this work provide insight in optimizing RIT protocols using radiolabeled agents with improved pharmacokinetics and biological half-lives.

## Introduction

Cancer represents a challenge in modern medicine, marked by its complex and heterogeneous nature, leading to significant morbidity and mortality worldwide^1^. Cancer stem cells (CSCs) are a subclass of cancer cells that can differentiate into various cell types and self-renew, similar to normal stem cells^2^. Recent investigations have shown that CSCs play a central role in the initiation, progression and resistance to cancer treatments. Unlike differentiated cancer cells (DCs), CSCs possess self-renewal and differentiation capabilities, which promote tumor heterogeneity and contribute to relapses^3–7^. Their plasticity allows them to adapt to the tumor microenvironment, while their resistance to conventional therapies, such as chemotherapy and radiotherapy, complicates the complete eradication of tumors. Due to these characteristics, CSCs have become major targets for the development of novel therapeutic strategies aimed at enhancing treatment efficacy and minimizing relapse^8–10^.

The clinical relevance of targeting CSCs in radioimmunotherapy (RIT) lies in their resistance to standard therapies, making them key contributors to cancer recurrence and metastasis. RIT is a targeted cancer treatment that combines radiation therapy with immunotherapy. This includes the use of monoclonal antibodies (mAbs) that are chemically conjugated to radioactive isotopes. The primary goal of RIT is to deliver a sufficient radiation dose to the tumor while minimizing damage to normal tissues such as bone marrow^11^. Optimizing RIT requires careful considerations of key parameters including total dose, initial dose rate and effective half-life (duration of irradiation)^12^. The choice of radionuclide plays a central role in determining the spatial range, energy deposition and biological effectiveness of RIT. Radionuclides commonly used in clinical pratice exhibit distinct emission types, energies and tissue penetration depths, resulting in different therapeutic profiles. Table 1 summarizes the main physical and radiobiological characteristics of the radionuclides considered in this study.

**Table 1:** Key characteristics of radionuclides in RIT.

Radionuclide	Emission type	Physical half-life	Max particle energy	Mean tissue range	Clinical applications	References
$^{90}\textrm{Y}$	$\beta ^{-}$(pure beta)	64.1 h	2.28 MeV	up to $\sim$ 11 mm	Radioembolization; liver tumors	^13,14^
$^{131}\textrm{I}$	$\beta ^{-} + \gamma$	$\sim$ 8 days	0.61 MeV($\beta$)	$\sim$ 2 mm	Differentiated tyroid cancer (therapy + imaging)	^15^
$^{177}\textrm{Lu}$	$\beta ^{-} + \gamma$	$\sim$6.7 days	0.497 MeV($\beta$)	$\sim$ 2 mm	Neuroendocrine tumors; $^{177}\textrm{Lu}$-DOTATATE	^16,17^
$^{225}\textrm{Ac}$	$\alpha$	$\sim$ 10 days	5.8–8.4.8.4 MeV($\alpha$)	0.05 - 0.1 mm	Targeted alpha therapy(for example PSMA prostate cancer)	^18^

For example, Jandl et al.^19^ described the effects of RIT targeting melanin with $^{188}\textrm{Re}$ labeled antibody and concluded that CSCs are killed at the same rate as melanoma cells. The authors also suggested that the results revealed that CSCs should be targeted directly with RIT by targeting cancer stem antigens on their surface. Giri *et al.*^20^ reported detailled timelines for CSC depletion in both human and canine experimental osteosarcoma models using $^{225}\textrm{Ac}$ and $^{177}\textrm{Lu}$ labeled RIT antibodies. The results showed that IF3 antibodies radiolabeled with $^{177}\textrm{Lu}$ or $^{225}\textrm{Ac}$ rapidly reduce IGF2R-positive osteosarcoma cells and gradually target CSCs, induce DNA damage ($\gamma$-H2AX) within 24 - 72 h, and modulate the tumor microenvironment by decreasing M2 macrophages. These results also showed that RIT acts differently across cell types, with $^{225}\textrm{Ac}$ being particularly effective against CSCs. Additionally, clinical challenges such as the slow uptake of radiolabeled mAbs by tumors and the variability of effective half-lives across different tissues, pose obstacles to therapeutic efficacy^21,22^. To address these challenges, it is essential to optimize the effective half-life of radiolabeled mAbs within tumors without adversely affecting vital organs^23^. Recent clinical data demonstrate that biological uptake and clearance. For example, the use of $^{177}\textrm{Lu}$ in targeted therapy has demonstrated notable efficacy in treating neuroendocrine tumors, due to well-calibrated dosimetry^24^. Similarly, studies have shown that well-adjusted protocols with radionuclides such as $^{131}\textrm{I}$ can limit side effects in patients with non-Hodgkin lymphoma^25^. Furthermore, if the biological half-life of mAbs in tumors exceeds that in normal tissues, radionuclides with extended physical half-lives could be advantageous by prolonging the action duration of radiolabeled mAbs within tumors^26,27^. Although RIT has shown potential in targeting tumor cells, the resistance of CSCs to conventional therapies poses a significant barrier to long-term treatment success, which makes the development of more effective, CSC-targeted strategies a crucial area of research. Researchers continue to explore innovative treatment modalities and RIT has emerged as a promising strategy, leveraging the specificity of antibodies to deliver targeted radiation to cancer cells^28,29^.

In cancer, microRNAs can act both as tumor suppressors and oncogenes, depending on the context. For example, miR-34a inhibits proliferation and promotes apoptosis in breast cancer but can act oncogenically in certain conditions^30^. In prostate cancer, miR-21 accelerates tumor progression when overexpressed, whereas underexpression may have a tumor-suppressive effect. Conversely, overexpressed miR-34a inhibits cancer cell proliferation, reduces tumor formation and targets CD44, limiting the renewal of CSCs and metastasis^31–33^. Recent studies also confirm miR-34a as an anticancer agent in prostate cancer, targeting CD44 to reduce metastasis^34,35^. These findings illustrate the dual roles of microRNAs in cancer, emphasizing the need for targeted therapies. In this study, we integrate the effects of microRNAs, particularly their delayed modulation of the differentiation between CSCs and DCs, into the mathematical framework to better reflect the biological timing of treatment resistance and recovery dynamics.

Mathematical modeling offers a powerful framework for optimizing therapeutic strategies and understanding the dynamics of CSC-targeted RIT. Although, mathematical models cannot fully replace in vitro experiments, they provide insights and, enable fast and cost-effective hypothesis testing^36^. Many mathematical CSCs models have been developed to simulate the various biological mechanisms, treatment responses and desease progression^37^. For example, Konstorum *et al.*^38^ demonstrated that feedback regulation in a CSC model can induce an allee effect. Olmeda and Amar^39^ explored clonal pattern dynamics in the context of CSCs, demonstrating that disordered patterns can be obtained inside a stable growing contour driven by the CSCs. In another study, Mori and Amar^40^ studied the role of stochasticity and drug effects in a dynamical model for CSCs. More recently, Essongo *et al.*^41^ developed a diffusive model for CSCs with time delay and radiotherapy effects, showing that CSC patterns can be completely eradicated before a critical time delay. However, existing mathematical models of CSCs have not incoporated the RIT effects and CSC oxygenation. This study aims to fill this gap by developing a mathematical model that integrates RIT effects, CSC dynamics and hypoxia. The main aim of this paper is to gain an insight into mechanisms of time evolution and spatiotemporal pattern formation in a tumor-immune-microenvironment-interaction model. We investigate both analytically and numerically a mathematical model of cancer cells under RIT, with particular emphasis on the behavior of CSCs and the hypoxia effects. To elucidate the impact of RIT on CSCs and microRNA-mediated interactions, we consider time delay in the model to capture the delayed response of cancer cells to therapeutic interventions mediated by microRNAs. This is crucial for understanding the dynamics of treatment response and resistance, as cancer cell may exhibit varying sensitivities to radiation over time. The analysis compares the physical half-lives of four radionuclides commonly used in RIT: $^{90}\textrm{Y}$, $^{131}\textrm{I}$, $^{177}\textrm{Lu}$ and $^{225}\textrm{Ac}$. In assessing the impact of these radionuclides on treatment duration, we aim to identify the optimal candidate for minimizing treatment duration while maximizing therapeutic efficacy.

We use the linear quadratic model, a sophisticated tool widely used in radiotherapy^42,43^, to generate biological effective dose (BED), tumor control probability (TCP) and survival fraction (SF) curves for each radionuclide. Although CSCs exhibit non-standard radiation responses due to their inherent radioresistance, the linear-quadratic model remains a valuable first-order approximation when appropriate radiosensitivity ratios are chosen to reflect CSC-specific radiosensitivity, as done in this study. Furthermore, in order to take into account the role of hypoxia or low oxygen levels in tumor progression and treatment resistance^44–47^, we investigate the hypoxia effects on the CSCs under RIT. We identify the radionuclide offering the shortest treatment duration while maintaining optimal therapeutic impact. This approach not only advances the understanding of the intricate mechanisms of cancer progression but also lays the groundwork for the development of new therapeutic strategies. We show that RIT can be applied before a cancer recurrence period in the dynamical system to achieve complete eradication of the tumor.

The rest of the paper is organized as follows. In section “Methods”, we present the diffusive cancer stem model with time delay and RIT, the oxygen effect modelling, the linear- quadratic model of RIT. In section "Stability and biomedical analysis of the equilibrium Points of model", we perform the stability analysis. In section "Results and discussion", the numerical results are presented and discussed. Section “Conclusion” concludes the paper.

## Methods

### Model equations

**Fig. 1:**
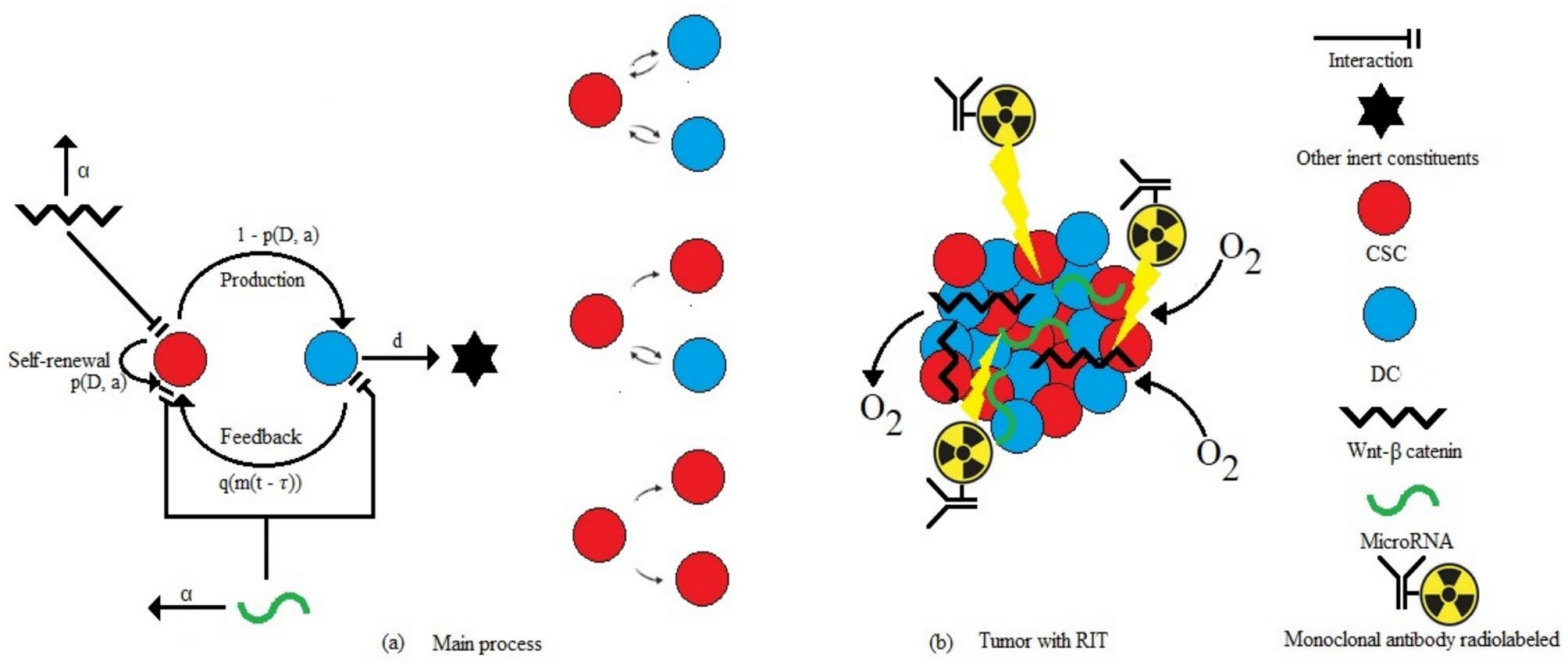
Schematic representation of model of CSCs: (**a**) main process and (**b**) tumor with RIT.

The initial model consists of a system of four non-linear differential equations describing the dynamics of three cell types^38–41^. The CSCs, which share characteristics with normal stem cells or progenitor cells, including self-renewal and multi-lineage differentiation abilities, leading to tumor growth and heterogeneity. The DCs, which are specific to the tumor and vary across organs. The inert constituents represent non-proliferative cells or passive components. CSCs drive cell division, promoting cell proliferation. The model also accounts for the removal of intermediate progenitors in the proliferation process. A CSC can undergo symmetric division, yielding two CSCs or two DCs, or asymmetric division, yielding one CSC and one DC. The average number of CSCs produced during division is regulated by proteins in the Wnt-$\beta$ catenin pathway^48^. Experimental data suggest the existence of a feedback mechanism triggered by phenotypic changes in DCs that revert to a CSC state, a phenomenon known as cancer cell plasticity. This process is mediated by activaton of microRNA molecules. The Fig. 1 describes the schematic representation of the CSCs model. The Fig. 1 (a) illustrates the main process of cell divisions and Fig. 1 (b) shows the CSCs model. Along the same line, the model considers time delay in microRNA-mediated dynamics and integrates RIT effects, thereby extending the CSC-specific framework of^41^ with radioimmunotherapy. This enhanced formulation provides a more comprehensive perspective on targeted cancer therapy, while building upon fundamental principles of time delay in biological regulation^49,50^. In this work, we take into account the effects of RIT in the model. This innovative therapeutic modality, combining antibodies targeting cancer cells with radioactive agents, offers new perspectives in cancer treatment. The effects of hypoxia are also considered, using the oxygen diffusion equation to model how oxygen levels vary within the tumor microenvironment and contribute to hypoxic conditions.

All the assumptions can be explained in the following set of equations:

1 $$\begin{aligned} \left\{ \begin{aligned} \frac{d S}{d t}&=(2p(D,a)-1)\varepsilon S + q(m(t-\tau ))D(t-\tau ) - \upsilon R_{imm}. S,\\ \frac{d D}{d t}&=2(1-p(D,a))\varepsilon S - (d+q(m))D { - \theta R_{imm}. D},\\ \frac{d a}{d t}&=a(\beta S\frac{a}{1+a} - \alpha ),\\ \frac{d m}{d t}&=\gamma e^{ -S/S_{0}} - \alpha m, \end{aligned} \right. \end{aligned}$$

where $S=S(t)$ is the concentration of CSCs, $D=D(t)$ is the concentration of DCs, $a=a(t)$ represents the concentration of Wnt-$\beta$ catenin pathway proteins and $m=m(t)$ the concentration of microRNAs. The quantity $p(D,a)=\frac{\eta a}{(1+\eta a)(1+\psi D)}$ denotes the probability of symmetric division producing two CSCs. The parameter $\eta$ refers to the definition of a probability coefficient *p*(*D*, *a*) for the inhibitor produced by *D* cells, where $\psi$ gives the intensity of the brake. The parameter $\varepsilon$ represents a time unit the inverse of the mitotic rate of the CSCs. The term $q(m(t-\tau ))=\frac{q_{0}}{2}\biggl (1+\tanh \biggl (\dfrac{m(t-\tau )-m_{0}}{\sigma }\biggr )\biggr )$ represents the DCs dedifferentiation rate. The parameter $q_0$ represents the maximum conversion rate per year of DCs into CSCs. Indeed, when *m* stimulates DCs that possess plasticity, the DCs transform into CSCs over a period of time. Given this process, we have considered a time delay $\tau$ which represents the time needed for DCs to produce CSCs after the interaction with microRNAs. The term $m_0$ is the minimal concentration of microRNAs. The parameter $\sigma$ measures the sensitivity of CSCs when interacting with microRNAs. The parameter $\upsilon$ is the CSCs death rate by radiation therapy. The term $R_{imm}$ represents the radioimmunotherapy function. The parameter *d* is the differentiated cancer cells death rate ($d>0$). The term $\theta$ is the DCs death rate by radiation therapy. Since CSCs are more radioresistant than DCs, the death rate $\theta$ was set higher than $\upsilon$. The parameter $\alpha$ represents the degradation rate of proteins and $\beta$ controller parameter of the aggressiveness of self-renewal Wnt-$\beta$ catenin pathway proteins. The term $\gamma$ represents the maximum concentration of microRNAs. The quantity $S_{0}$ is a very low fraction of *S* of the entire tumor population. In practice when *S* is below $S_{0}$, the activator *m* starts to grow as *q*(*m*), leading to an increase of *S*. The above considerations can facilitate a comprehensive understanding of tumor biology, the development of more effective therapeutic strategies, and more precise prediction of clinical outcomes. The units of variables and parameters used in this paper are given in the Table 2. The radioimmunotherapy function defined by the following equation^51^:

2 $$\begin{aligned} R_{imm} = (\alpha _{c}\mathcal {D})\times RE, \end{aligned}$$

with *RE* being the relative effectiveness per unit dose and is given following the derivation in^43^ by

3 $$\begin{aligned} RE= 1+\frac{r_{0}}{ln2}\varLambda , \end{aligned}$$

where

$$\begin{aligned} \varLambda = \frac{2T_{\mu }^{4}(T_{e}-T_{eu})}{(T_{\mu }^{2}-T_{e}^{2})(T_{\mu }^{2}-T_{eu}^{2})}+ \frac{2 T_{e}T_{eu}T_{\mu }}{(T_{e}^{2}-T_{eu}^{2})} \biggl ( \frac{T_{e}}{T_{\mu }-T_{e}}+\frac{T_{eu}}{T_{\mu }-T_{eu}}\biggr )-\frac{T_{\mu }}{T_{e}-T_{eu}}\biggl (\frac{T_{e}^{2}}{T_{\mu }-T_{e}}+\frac{T_{eu}^{2}}{T_{\mu }-T_{eu}} \biggr ). \end{aligned}$$

Note that $T_{e}$ is greater than $T_{eu}$. The effective clearance half-time $T_{e}$ is given by $T_{p}T_{b}/(T_{p}+T_{b})$ and the effective uptake half-time $T_{eu}$ is given by $T_{p}T_{u}/(T_{p}+T_{u})$, where $T_{u}$ and $T_{b}$ are respectively the biological uptake half-time and the clearence half-time, $T_{p}$ the physical half-life and $T_{\mu }$ the repair half-time. The quantity $r_{0}$ is the extrapolated initial dose rate^12^. The quantity $\mathcal {D}$ denotes the total dose.

In this study, radiosensitivity parameters $\alpha _{c}$ and $\beta _{c}$ for CSCs and DCs were set to $\alpha _{c}$ = 0.46 Gy$\phantom{0}^{-1}$ and $\beta _{c}$ = 0.30 Gy$\phantom{0}^{-2}$, based on experimental and theoretical studies of target radionuclide therapy^52–54^. Table 2 summarizes the variables and parameters in Eq. 1.

**Table 2:** Variables and parameters of Eq. 1.

Variable	Description	Unit	reference
*S*	concentration of CSCs	$\mathrm {cell.mm^{-3}}$	^41^
*D*	concentration of DCs	$\mathrm {cell.mm^{-3}}$	^41^
*a*	concentration of Wnt-$\beta$	$\mathrm {molecule.mm^{-3}}$	^41^
*m*	concentration of microRNAs	$\mathrm {molecule.mm^{-3}}$	^41^
Parameter	Description	Value & Unit	reference
$\eta$	positive feedback strength of Wnt-$\beta$	1 $\mathrm {mm^{3}.molecule^{-1}}$	^41^
$\psi$	negative feedback strength of DCs	0.5 $\mathrm {mm^{3}.cell^{-1}}$	^41^
$\varepsilon$	mitotic rate of the CSCs	1 $\mathrm {year^{-1}}$	^41^
$$q_{0}$$	maximum DCs dedifferentiation rate	$$q _{0}$$$$> d$$$$\mathrm {year^{-1}}$$	^39^
$\tau$	DC-to-CSC conversion time	$\textrm{year}$	^41^
$m_{0}$	minimal concentration of microRNAs	0.05 $\mathrm {molecule.mm^{-3}}$	^39^
$\sigma$	sensitivity of CSCs when interacting with microRNAs	0.05 $\mathrm { molecule.mm^{-3}}$	^41^
$\upsilon$	CSCs death rate by radiation therapy	25 $\mathrm {year^{-1}}$	^41^
*d*	DCs death rate	36.5–109.5.5.5 $year^{-1}$	^40^
$\theta$	DCs death rate by radiation therapy	50 $\mathrm {year^{-1}}$	^55^
$\alpha$	degradation rate of proteins or molecules	0.3 $\mathrm {mm^{3}.molecule^{-1}}$	^41^
$\beta$	aggressiveness of self-renewal Wnt-$\beta$ rate	1 $\mathrm {mm^{3}.cell^{-1}.year^{-1}}$	^41^
$\gamma$	maximum concentration of microRNAs	1 $\mathrm {molecule.mm^{-3}.year^{-1}}$	^41^
$S_{0}$	minimal concentration of CSCs	0.038 $\mathrm {cell.mm^{-3}}$	^41^

### The oxygen effect: mathematical modeling

It is known that the cancerous cells with low oxygen conditions (hypoxia) exhibit radioresistance compared to those with higher oxygen levels^56^. The oxygen effect in radiology is a phenomenon where the presence of oxygen enhances the damage caused by radiation therapy to cancerous cells. This effect is quantified using the oxygen enhancement ratio (OER), which is defined as the ratio of doses required to achieve the same level of biological damage under hypoxic (for an oxygen partial pressure $P_{o}$) and normoxic (normal oxygen) conditions^57^. Following the work^58^, the distributions of oxygen partial pressure were calculated by solving the oxygen reaction-diffusion equation:

4 $$\begin{aligned} \frac{\partial P_{ o}}{\partial t} = - \frac{C_{max}.P_{o}}{k+P_{o}} { (S + D) } + D_{O_{2}}\Bigl (\frac{\partial ^{2}P_{o}}{\partial x^{2}}+\frac{\partial ^{2}P_{o}}{\partial y^{2}}\Bigr ). \end{aligned}$$

The oxygenation model Eq. (4) describes oxygen dynamics in short period of time $t'$ (seconds). $P_{o}=P_{o}(x, y, t')$ is the oxygen partial pressure at position (*x*, *y*) on a two-dimensional square domain of width $l=20$ mm subject to the Dirichlet boundary conditions $P_{o}(x, y, t')= P_{c} = 40$ mmHg which represent implicitly a source of oxygen at the surface of capillaries. $D_{O_{2}}$ is the diffusion coefficient of oxygen, $C_{max}$ is the maximum metabolism, which represents the maximum available oxygen partial pressure within the tumor region and is used to describe the oxygenation level accessible to cancer cells^59^. In this study, the value of $C_{max}$ is compared to the typical hypoxic conditions observed in solid tumors. Biologically, $D_{O_{2}}$ = 0.002 $\mathrm {mm^{2}.s^{-1}}$ and $C_{\max }$ = 15 $\mathrm {mmHg.s^{-1}}$. $S=S(t)$ and *D*(*t*) the concentration of CSCs and DCs respectively, *k* is the partial pressure at which metabolism reaches half its maximum value, all the values of these quantities are given in table 3.

**Table 3:** Values of the parameters of Eq. (4).

Parameter	Value/unit	Reference
$C_{max}$	15 mmHg. mm$\phantom{0}^{3}$. $\hbox {cell}^{-1}$. s $\phantom{0}^{-1}$	^58^
*k*	2.5 $\textrm{mmHg}$	^58^
$D_{O_{2}}$	0.002 mm$\phantom{0}^{2}$. s $\phantom{0}^{-1}$	^60^

### The linear quadratic model for RIT

The linear quadratic model commonly employed in studying the effects of radiation, was initially developed as a practical tool to understand how radiation affects chromosome damage and slows down the growth of broad beans^42,61,62^. However, it also has a strong biophysical basis taking into account how radiation damage builds up from both lethal events and the combined effects of less severe incidents^63^. The dose rate in the tumor decreases mono-exponentially from an initial value of $r_{0}$ in brachytherapy using permanent sealed source implants. This is not the case for therapeutic strategies that implement internally administered radionuclides, such as radioimmunotherapy. In the latter case, the dose rate in the tumor increases from an initial value of zero to a maximum value as the radioactivity is taken up by the tumor, then decreases asymptotically to zero as the radioactivity is eventually cleared from the tumor. Generally, the function of the dose rate to tumor or critical organs is represented by^51,64–66^

5 $$\begin{aligned} r(t)= r_{0}(e^{-ln2 t/T_{e}}-e^{-ln2 t/T_{eu}}). \end{aligned}$$

The SF of clonogens in a voxel with capillary density $\nu _{c}$ receiving an extrapolated initial dose $r_{0}$ can be described by the linear-quadratic model modified to account for OER^67^:

6 $$\begin{aligned} SF(r_{0}, \nu _{c}) = \int _{0}^{\infty }dP_{o} f(P_{o}, \nu _{c})exp(-R_{imm}), \end{aligned}$$

where $f(P_{o}, \nu _{c})$ represents the unit-normalized probability density function of the oxygen partial pressure $P_{o}$ at a point in space with capillary density $\nu _{c}$, and

7 $$\begin{aligned} R_{imm}=(\alpha _{c}\mathcal {D})\times RE =\alpha _{c} \times BED. \end{aligned}$$

Here, the concept of the biological effective dose (*BED*) is used to incorporate the effects of extrapolated initial dose $r_{0}$ and repair mechanisms in RIT. Combining the oxygen effect with *BED* modeling provides a comprehensive framework to predict the effectiveness of RIT under varying oxygen conditions. Thus, the modified *BED* that includes the oxygen effect is:

8 $$\begin{aligned} BED(P_{o}, r_{0}) = \left\{ \begin{aligned}&\frac{\mathcal {D}}{OER(P_{o})}\biggl (1+\frac{r_{0}}{ln2(\alpha _{c}/\beta _{c})OER(P_{o})}.\varLambda \biggr )-ln2\frac{(T-T_{k})}{\alpha _{c}T_{d}}, \hspace{25pt} T> T_{k}\\&\frac{\mathcal {D}}{OER(P_{o})}\biggl (1+\frac{r_{0}}{ln2(\alpha _{c}/\beta _{c})OER(P_{o})}.\varLambda \biggr ) \hspace{90pt} T \le T_{k}, \end{aligned} \right. \end{aligned}$$

where $T_{d}$ is the proliferation doubling time, *T* is the overall treatment time, and $T_{k}$ is the off-set time for repopulation to initiate^68^. The total dose $\mathcal {D}$ to the tissue is obtained by integration of Eq. (5) from 0 to $\infty$ for complete decay yields the total dose $\mathcal {D}$ as follows

9 $$\begin{aligned} \mathcal {D}=\frac{r_{0}}{ln2}\tau _{e}, \end{aligned}$$

where $\tau _{e}= T_{e}-T_{eu}$ is the effective time.

The partial pressure dependence of the oxygen enhancement ratio was approximated as^57^

10 $$\begin{aligned} OER(P_{o})=\frac{OER_{max}(P_{o}+K_{m})}{OER_{max}.P_{o}+K_{m}}, \end{aligned}$$

where $OER_{max}$ the maximum *OER* value and $K_{m}$ is the partial pressure at which *OER* achieves half its maximum value.

#### The tumor control probability (TCP)

The TCP is a central parameter for evaluating cancer treatment efficacy. Generally, a higher TCP correlates with more favorable patient outcomes and extented survival. However, multiple factors can affect the TCP. A primary factor is the cancer stage, representing the extent of disease progression. Tumors at earlier stages usually exhibit higher TCP compared to those at advanced stages. Tumor location is another significant variable, as tumors in sensitive or anatomically challenging areas may limit treatment effectiveness^69^. The type of therapy administered is also critical to TCP outcomes. Different treatments, such as surgery, radiotherapy and chemotherapy may have different effects on the tumor control probability^41^. Additionally, individual patient responses are pivotal, as variability in biological reactions to treatment can significantly influence TCP. The TCP, quantitatively represents the probability of avoiding local recurrence at total dose $\mathcal {D}$. The application of Poisson statistics and the linear-quadratic model incorporating the Poissonian probability^70,71^ lead to

11 $$\begin{aligned} TCP=exp(-N.SF(r_{o}, \nu _{c}))=exp(-Nexp(-\alpha _{c} BED(P_{o}, r_{o}))), \end{aligned}$$

where $N=\varsigma .V$ ($\varsigma$ is cell concentration and *V* is the tumoral volume) represents the initial number of potential proliferating cells in tumor and *SF* is the cell survival probability such that $SF(r_{0}, \nu _{c}) = \int _{0}^{\infty }dP_{o} f(P_{o}, \nu _{c})exp(-R_{imm})$. The closer TCP is to one, the greater the probability that all cancer stem cells die out and the tumor is controlled^72^. Spoormans *et al.*^68^ discussed that TCP takes into account the total absorbed dose and the radiosensitivity of the tumour tissue, while distinguishing between repairable and sublethal single-strand DNA breaks and irreparable and lethal double-strand DNA breaks. The parameters related to radiotherapeutic values are given in the Table 4.

**Table 4:** Values of the radiobiological parameters.

Parameter	Value/unit	References
$\alpha _{c}$	0.46 $\mathrm {Gy^{-1}}$	^52–54^
$\beta _{c}$	0.30 $\mathrm {Gy^{-2}}$	^52–54^
$T_{\mu }$	0.062 *day*	^73^
$OER_{max}$	3	^57,74^
$K_{m}$	3.28 mmHg	^75^
$T_{b_{^{90}Y}}$	8.00 days	^11,76^
$T_{b_{^{131}I}}$	2.50 days	^77^
$T_{b_{^{177}Lu}}$	3.50 days	^77^
$T_{b_{^{225}Ac}}$	9.50 days	^76^
$T_{u_{^{90}Y}}$	3.00 days	^78^
$T_{u_{^{131}I}}$	1.20 day	^51,79^
$T_{u_{^{177}Lu}}$	1.15 day	^80^
$T_{u_{^{225}Ac}}$	3.20 days	^81^

## Stability and biomedical analysis of the equilibrium points of model

Equation 1, without a RIT function has three equilibrium points:

$E^{(1)}=(0,0,0,m_{1})$ with $S=0,D=0, a=0$ and $m_{1}$ an arbitrary value.

$E^{(2)}=(S_{2},D_{2},0,m_{2})$ with $S_{2}=-S_{0}Log\biggl (\frac{\alpha m_{2}}{\gamma }\biggr ), D_{2}=\frac{S_{2}}{d}, a_{2}=0$ and $m_{2}$ an arbitrary value.

$$E^{(3)}=(S_{3},D_{3},a_{3},m_{3})$$ with $$S_{3}=\frac{\alpha (1+a_{3})}{\beta a_{3} }, D_{3}=\frac{\alpha (1+a_{3})}{\beta d a_{3}}, m_{3}=\frac{\gamma }{\alpha } e^{-S_{3}/S_{0}}$$ and $$a_{3}=\frac{1+{\frac{\psi \alpha }{d\beta }}(1+\eta )}{2\eta (1-{\frac{\psi \alpha }{d\beta }})}(1+\sqrt{1+\frac{4{\frac{\psi \alpha }{d\beta }}\eta (1-{\frac{\psi \alpha }{d\beta }})}{(1+{\frac{\psi \alpha }{d\beta }}(1+\eta ))^{2}}})$$.

The equilibrium point $E^{(1)}=(0,0,0,m_{1})$ is characterized by the absence of cancer stem cells (CSCs), differentiated cancer cells (DCs), and Wnt-$\beta$ catenin signaling activity, with $m_{1}$, representing microRNA concentration levels, being arbitrary. $E^{(1)}$ represents a theoretical state where no cancerous activity exists. This is crucial as it provides a baseline scenario, which could be described as an ideal or disease-free state. Achieving $E^{(1)}$ would imply the complete eradication of cancer cells. However, this is not feasible in a real biological system due to the persistent nature of cancer cells and their ability to evade complete destruction. Thus, while $E^{(1)}$ serves as a conceptual goal, it remains practically unattainable with current therapeutic methods. Moreover, the importance of $E^{(1)}$ lies in its function as a benchmark for evaluating the effectiveness of cancer therapies. Even though the most aggressive treatments typically leave some residual cancer cells, comparing these outcomes to $E^{(1)}$ helps in assessing the success of these interventions. Consequently, understanding $E^{(1)}$ is essential for developing strategies aimed at reducing cancer activity to the lowest possible level. Additionally, $E^{(1)}$ underscores the ultimate aim of cancer treatment, which is to minimize the presence of cancerous cells. Despite being a theoretical construct, it drives the pursuit of more effective therapies that aspire to come as close as possible to this ideal state. $E^{(1)}$ serves as a crucial reference point, guiding the development of treatment protocols and helping to measure their success in clinical settings.

The equilibrium point $E^{(2)}=(S_{2},D_{2},0,m_{2})$ describes a state where both CSCs $(S_{2})$ and DCs $( D_{2})$ are present, but the Wnt-$\beta$ catenin signaling pathway $( a_{2})$ remains inactive. The levels of CSCs and DCs concentrations are influenced by the microRNA level $( m_{2})$, which is arbitrary but within specific constraints. $E^{(2)}$ is significant as it represents a controlled state of cancer, where the disease is present but not aggressive. Specifically, the inactivity of the Wnt-$\beta$ catenin signaling pathway implies that the signaling mechanisms driving uncontrolled proliferation are suppressed. Consequently, this equilibrium point aligns with clinical scenarios where cancer is kept in check, often through targeted therapies that modulate signaling pathways.

Moreover, $E^{(2)}$ highlights the potential of microRNAs as therapeutic agents. By influencing the stability of this equilibrium, microRNAs can be targeted to maintain a non-aggressive cancer state. Thus, therapies designed to adjust microRNA levels could be pivotal in managing cancer more effectively. Additionally, the stability of $E^{(2)}$ suggests that it is possible to sustain this equilibrium under certain conditions, such as ensuring $( d < q_{0})$ and $( m_{2} < \frac{\gamma }{\alpha })$. This insight is crucial for developing treatments that aim to stabilize cancer at this controlled state. From a therapeutic perspective, $E^{(2)}$ represents an optimal target. Maintaining the system at this equilibrium prevents progression to more aggressive forms of cancer. Therefore, treatment strategies that inhibit the Wnt-$\beta$ catenin signaling pathway and regulate microRNA levels are essential in achieving and maintaining this state. Consequently, $E^{(2)}$ serves as a practical goal for clinical interventions aimed at long-term cancer management. Additionally, $E^{(2)}$ underscores the importance of early intervention and consistent monitoring in cancer treatment. By understanding the parameters that keep the system at this equilibrium, clinicians can tailor therapies to prevent deviations towards more harmful states. This not only enhances patient outcomes but also reduces the likelihood of recurrence and progression.

The equilibrium point $E^{(3)}=(S_{3},D_{3},a_{3},m_{3})$ is characterized by active Wnt-$\beta$ catenin signaling pathway $( a_{3})$, leading to the presence of both CSCs $(S_{3})$ and DCs $( D_{3})$, and specific microRNA levels $( m_{3})$ influenced by system parameters. $E^{(3)}$ signifies a state of uncontrolled cancer proliferation, which is particularly critical as it represents an advanced and aggressive phase of the disease. The activation of the Wnt-$\beta$ catenin pathway at this point is a hallmark of many cancers, driving rapid and uncontrolled cell growth. Consequently, $E^{(3)}$ aligns with clinical observations where cancer becomes highly resistant to standard treatments and more difficult to manage. Moreover, the instability associated with $E^{(3)}$ suggests that once cancer reaches this state, it tends to worsen. This is significant for clinicians as it highlights the need for early intervention to prevent the system from evolving to this aggressive equilibrium. Therefore, aggressive therapeutic strategies are required to disrupt the Wnt-$\beta$catenin signaling pathway, which is central to maintaining $E^{(3)}$. However, $E^{(3)}$ shows the complexity of treating advanced cancer. Since CSCs are a key component of this equilibrium, therapies need to specifically target these cells to be effective. This includes strategies that either eliminate CSCs or induce their differentiation into less harmful cell types. Thus, the biomedical interpretation of $E^{(3)}$ points to the necessity of multi-faceted treatment approaches that address both the signaling pathways and the cellular composition of the tumor. Furthermore, targeting the Wnt-$\beta$ catenin pathway is crucial as its inhibition could potentially revert the system to a less aggressive state, such as $E^{(2)}$. Therefore, drugs that specifically inhibit this pathway are essential in managing advanced cancer. Consequently, understanding $E^{(3)}$ not only helps in grasping the dynamics of aggressive cancer but also in designing effective treatment protocols aimed at reverting or stabilizing the disease. Additionally, $E^{(3)}$ provides insights into the mechanisms of cancer metastasis and resistance. The activation of Wnt-$\beta$catenin signaling is often associated with these processes, suggesting that targeting this pathway could also mitigate metastatic spread and reduce resistance to therapy. This further emphasizes the importance of advanced research and development in creating targeted inhibitors for this pathway.

## Results and discussion

In this section, we present the results related to the effectiveness of RIT. We conduct numerical simulations to evaluate the dynamics of the system, using equations for cell and tissue oxygenation with parameters relevant to prostate cancer. All simulations are performed in MATLAB and a system size of $20\times 20$ spatial units is used for Eq. (4). The numerical integration of Eqs. (1) and (4) is done via the fourth-order Runge-Kutta method with Hermite interpolation to ensure precision and stability compared to other numerical methods^82,83^. In these simulations, to avoid numerical stiffness, we use a time step $\Delta t=0.01$ for equation (1) and the time step $\Delta t=0.01$ and spatial steps $\Delta x=\Delta y=0.2$ are used for Eq. (4), under Dirichlet’s boundary conditions. For the initial conditions, we introduce small inhomogeneous perturbation from homogeneous equilibrium state $\hbox {E}^{(2)}$. The initial distribution of the oxygen pressure is given by $P_{O}(x, y, 0)= P_{O_{initial}}+(0.1cos((x^{2}+y^{2}-55)\pi -0.01sin((x^{2}+y^{2}+91)\pi )))$, where $P_{O_{initial}}=40$ and the other initial conditions can be found in^41^. In addition to these simulations, we also perform a sensitivity analysis of the parameters to assess their influence on tumor dynamics and treatment outcomes.

### The sensitivity analysis

**Fig. 2:**
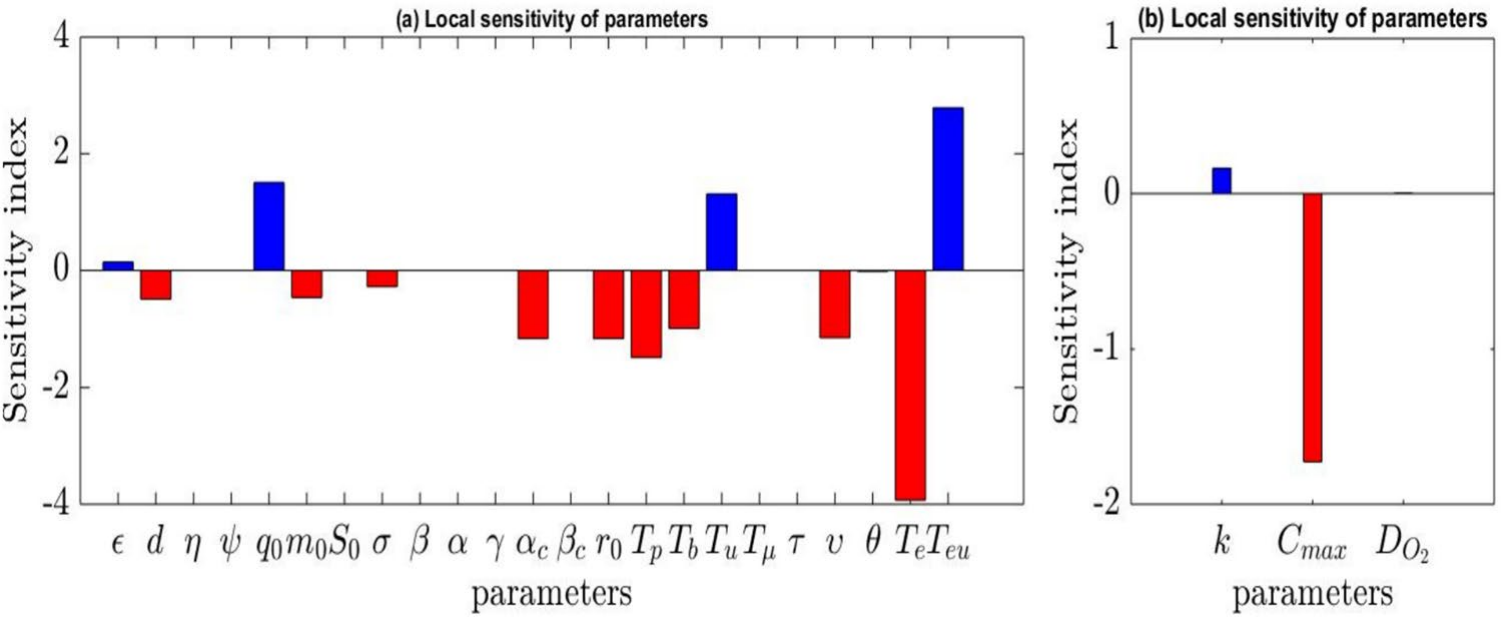
Snapshots of (**a**) the local sensitivity analysis of the principal parameters in Eq. (1) at t = 16 years when $\tau$ = 8.8964 years $< \tau _{c}$ = 11.8964 years and $r_{0}$ = 50 $\mathrm {Gy.year^{-1}}$; (**b**) the local sensitivity analysis of the principal parameters in Eq. (4) at *t* = 2 seconds when the CSC and DC concentration $(S + D)$ = 1 $\mathrm {cell.mm^{-3}}$.

The numerical local one-at-a-time (OAT) sensitivity analysis was performed to evaluate the influence of biological and pathophysiological parameters on CSC dynamics and tumor oxygen pressure. Each parameter was individually perturbed by +0.1% of its nominal value, while all other parameters were kept fixed. Sensitivity indices were computed using finite-difference approximations of the corresponding outputs of the system of equations. For Eq. (1), the output used to compute the sensitivity indices corresponds to the final CSC concentration, where diffusion was neglected, with the therapy initiated at $t=\tau$ = 8.8964 years and the final simulation time *t* = 16 years. For the Eq. (4) (Fig. 2), which includes diffusion, the output corresponds to the tumor oxygen pressure, with the final simulation time *t* = 2 seconds. The normalized sensitivity indices reported in Table 5 (for Eq. (1)) and Table 6 (for Eq. (4)) are ranked in decreasing order of their absolute values, highlighting the most influential parameters. Positive and negative signs indicate whether an increase or decrease of the corresponding output, respectively.

**Table 5:** Normalized local sensitivity indices for Eq. (1).

Parameter	Sensitivity index
$T_e$	−3.9305445601
*Teu*	+2.7880592573
$q_0$	+1.5066648866
*Tp*	−1.4818897145
*Tu*	+1.3102969081
$r_0$	−1.1587808595
$\alpha _c$	−1.1587801892
$\upsilon$	−1.1437205781
*d*	−0.4910930499
$m_0$	−0.4631907212
$\sigma$	−0.2775354758
$\epsilon$	+0.1443354156
$\theta$	−0.0150771902
$T_{\mu }$	−0.0000353946
$\tau$	+0.0000317816
$\alpha$	−0.0000192055
$\eta$	+0.0000083215
$\beta _c$	−0.0000003355
$S_0$	+0.0000001567
$\gamma$	+0.0000000656
$\beta$	+0.0000000018
$\psi$	−0.0000000004

**Table 6:** Normalized local sensitivity indices for Eq. (4).

Parameter	Sensitivity index
$C_{\max }$	−1.7261084453
*k*	+0.1628999671
$D_{O_2}$	+0.0034314747

For Eq. (1), the sensitivity analysis reveals that several biological and therapeutic parameters strongly influence CSC dynamics. The effective half-life of clearance $T_{e}$ and the effective half-life of uptake $T_{eu}$ exhibit the highest absolute sensitivity indices, indicating that radionuclide pharmacokinetics play a dominant role in treatment efficacy. Variations in these parameters strongly affect CSC survival, emphasizing the importance of personalized dosimetry in radionuclide therapy. Similarly, the physical and biological half-lives $T_{b}$ and $T_{u}$ significantly impact CSC dynamics. This suggests that optimizing radionuclide uptake and clearance rates may substantially improve therapeutic outcomes. The radiosensitivity parameters $\alpha _{c}$ and $\beta _{c}$, together with the initial radiation dose $r_{0}$, reflect the ability of CSCs to respond to radiation-induced damage. Their high sensitivity highlights the importance of adapting radiation protocols to CSC-specific radiobiological properties. The treatment-related parameter $\upsilon$ also shows strong sensitivity, indicating that treatment intensity plays a crucial role in controlling CSC populations. Appropriate adjustment of treatment strength may therefore enhance tumor control while limiting adverse effects. The mitotic rate of CSCs $\varepsilon$ presents a moderate positive sensitivity, suggesting that increased CSC proliferation contributes to tumor persistence. Targeting CSC self-renewal may thus represent a promising therapeutic strategy. The death rate of DCs *d* shows moderate sensitivity, indicating that increasing DC apoptosis may contribute to tumor control. Although, its effect remains limited compared to CSC-targeted mechanisms. The parameters $m_{o}$ and $\sigma$ highligth the influence of microRNA-mediated pathways on CSC plasticity. Increasing microRNA activity may reduce CSC self-renewal and promote differentiation, thereby limiting tumor aggressiveness. The parameter $\psi$ describing the influence of DCs on CSCs, shows a low sensitivity value. Nevertheless, its biological role remains important, as interactions between DCs and CSCs contribute to regulating tumor growth. A reduction in this interaction may weaken inhibitory feedback, whereas strengthening it could help stabilize tumor dynamics. The conversion rate $q_{0}$ exhibits significant sensitivity, suggesting that phenotypic plasticity through dedifferentiation contributes to CSC maintenance but is not the dominant mechanism. Other parameters, including $\eta , \beta , \gamma$ and $\tau$ display very low sensitivity indices, indicating a limited influence on CSC dynamics under the considered parameter range.

For Eq. (4), the maximum oxygen partial pressure $C_{max}$ exhibits the highest sensitivity magnitude, indicating that oxygen availability strongly affects tumor oxygen levels. Clinically, this suggests that improving vascular perfusion and oxygen delivery may enhance treatment response. The parameter of partial pressure at half-maximum metabolism *k* presents moderate sensitivity. It indicates that variations in this parameter significantly influence the tumor oxygenation. A higher value of *k* implies increased oxygen requirements for cellular metabolism, which may limit tumor survival under hypoxic conditions. The oxygen diffusion Coefficient $D_{O_{2}}$ shows weak positive sensitivity, indicating a limited influence on the mean tumor oxygen pressure. It suggests that variations in oxygen transport efficiency have a relatively minor effect on global oxygenation compared to metabolic parameters. A higher value slightly enhances oxygen penetration into tumor tissue, which may marginally improve oxygen homogeneity and therapeutic response.

In summary, the sensitivity analysis identifies the key parameters governing CSC dynamics and tumor oxygenation. For Eq. (1), radionuclide pharmacokinetics and radiosensitivity parameters predominantly control the final CSC concentration, whereas for Eq. (4), oxygen availqbility and metabolic demand mainly determine the tumor oxygen pressure. These results provide useful guidance for optimizing therapeutic strategies. However, the OAT method remains limited by its local nature. Its inability to capture parameter interactions and its dependence on nominal parameter values. Global sensitivity analysis, which takes into account simultaneous variations of multiple parameters, will be addressed in future work for a better understanding of parameter interplay and influence on model outputs.

### The results of simulations without the oxygen effects

In this section, we present the obtained simulation results without oxygen effects. All the simulations are performed using BED, TCP. The parameter values for physical and biological half-lives, as well as the biological uptake and clearance half-lives of the radiolabeled antibodies, are provided in Table 4.

**Fig. 3:**
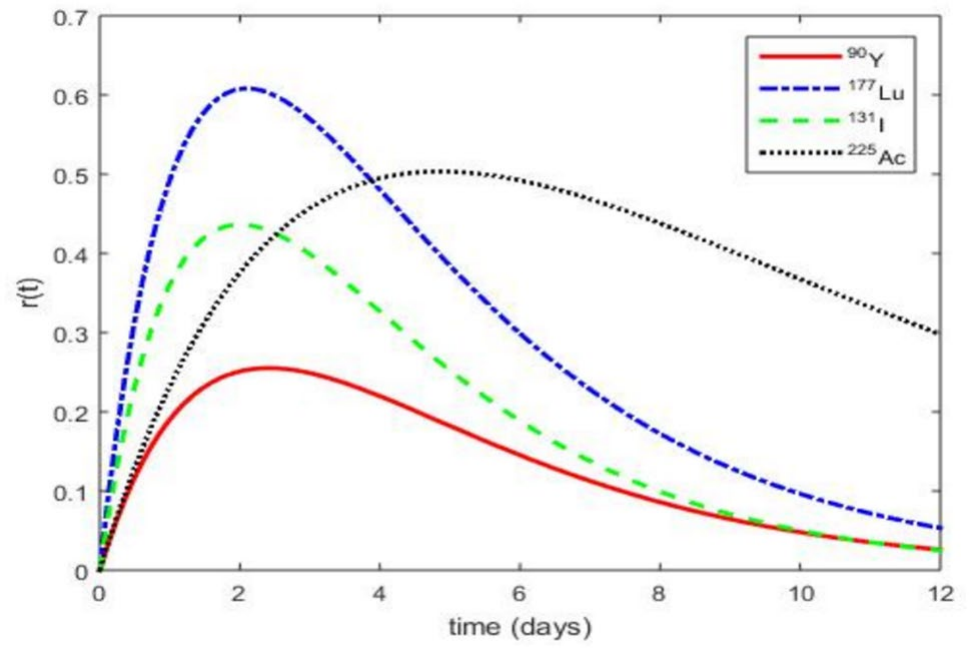
Evolution of dose rate *r*(*t*) for different radionuclides as a function of the time *t* when the extrapolated initial dose $r_{0}$ = 2 $\mathrm {Gy.day^{-1}}$ and the other parameters values are given in the Table 4.

We plot in Fig. 3 the function *r*(*t*) given by Eq. (5). Figure 3 illustrates that each radionuclide exhibits a distinct kinetic profile: $^{131}\textrm{I}$ exhibits the earliest peak at $t=1.939$ $\textrm{day }$ with a maximum dose rate of 0.4362 $\mathrm {Gy.day^{-1}}$. $\mathrm {^{177}Lu}$ peaks at $t=2.061$ $\textrm{day}$, reaching 0.6082 $\mathrm {Gy.day^{-1}}$, indicating an intermediate balance between early dose delivery and moderate persistence. $^{90}Y$ shows an early peak at $t=2.424$ $\textrm{day}$, with a maximal dose rate of 0.2552 $\mathrm {Gy.day^{-1}}$, reflecting rapid but short-lived exposure. In contrast, $^{225}\textrm{Ac}$ reaches its maximum dose rate at $t=4.848$ $\textrm{day}$, with a peak of 0.5033 $\mathrm {Gy.day^{-1}}$, lower than that of $^{177}\textrm{Lu}$ but delivered over a substantially longer timescale, indicating a slower and more prolonged therapeutic action.

These profiles directly impact therapeutic efficacy against CSCs, which are characterized by low proliferative activity, therapy resistance, and residence in poorly vascularized hypoxic niches. Fast-acting isotopes such as $^{90}\textrm{Y}$ may rapidly debulk DCs, but their transient nature limits CSC penetration. The simulations confirm that $^{90}\textrm{Y}$ fails to eliminate CSCs, often allowing tumor regrowth. Conversely, $^{225}\textrm{Ac}$, despite its moderate peak intensity, maintains a sustained dose delivery compatible with the prolonged cell cycle times of CSCs. Its alpha emissions deliver highly localized, potent cytotoxicity, even in hypoxic conditions, leading to efficient CSC depletion. $^{177}\textrm{Lu}$ offers intermediate efficacy, while $^{131}\textrm{I}$ shows only partial therapeutic benefits. The magnitude and timing of *r*(*t*) peaks are crucial indicators of therapeutic potential. These results underscore that long-acting alpha emitters such as $^{225}\textrm{Ac}$ are optimally suited for CSC-targeted cancer therapy, aligning pharmacokinetic profiles with biological tumor resilience.

**Fig. 4:**
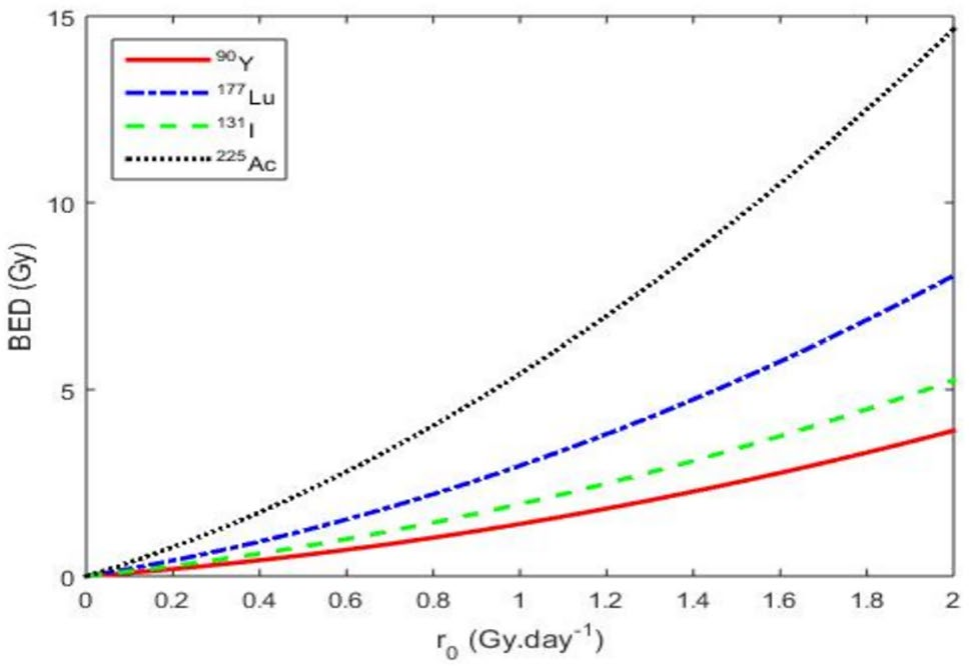
Evolution of BED as a function of the extrapolated initial dose $r_{0}$ using the parameter values from Table 4.

From the second Eq. (8) without the $\mathrm {OER(P_{0})}$, we plot in Fig. 4 the BED curve. Figure 4 illustrates a distinct hierarchy in BED profiles. $^{225}\textrm{Ac}$ achieving the highest values, followed by $^{177}\textrm{Lu}$, $^{131}\textrm{I}$, and $^{90}\textrm{Y}$. This ranking reflects differences in physical half-lives, biological retention, and radiation quality.

$^{225}\textrm{Ac}$, an alpha-emitting radionuclide with high linear energy transfer (LET), exhibits a prolonged physical half-life of approximately 10 days and slow biological clearance, supporting sustained intratumoral irradiation. These features render it particularly suited for eradicating therapy-resistant subpopulations such as cancer stem cells. $^{177}\textrm{Lu}$ offers a balanced profile, combining a moderate half-life (6.7 days) with reasonable tumor retention. Its lower LET is compensated by its compatibility with molecular targeting platforms, notably PSMA-directed ligands in prostate cancer. $^{131}\textrm{I}$, despite its long physical half-life (8.02 days), displays rapid biological clearance, reducing its intratumoral residence time. This limits its efficacy in poorly perfused tumor regions or stem-cell-enriched niches. $^{90}\textrm{Y}$, a high-energy beta emitter, shows the lowest BED due to its short half-life (2.67 days) and limited biological retention, leading to suboptimal exposure in slowly proliferating tumors. Collectively, these data underscore the biological and therapeutic advantages of alpha emitters such as $^{225}\textrm{Ac}$ in scenarios demanding durable, high-LET irradiation. Optimal radionuclide selection should integrate both dosimetric considerations and biological parameters such as clearance kinetics and microenvironmental penetration.

**Fig. 5:**
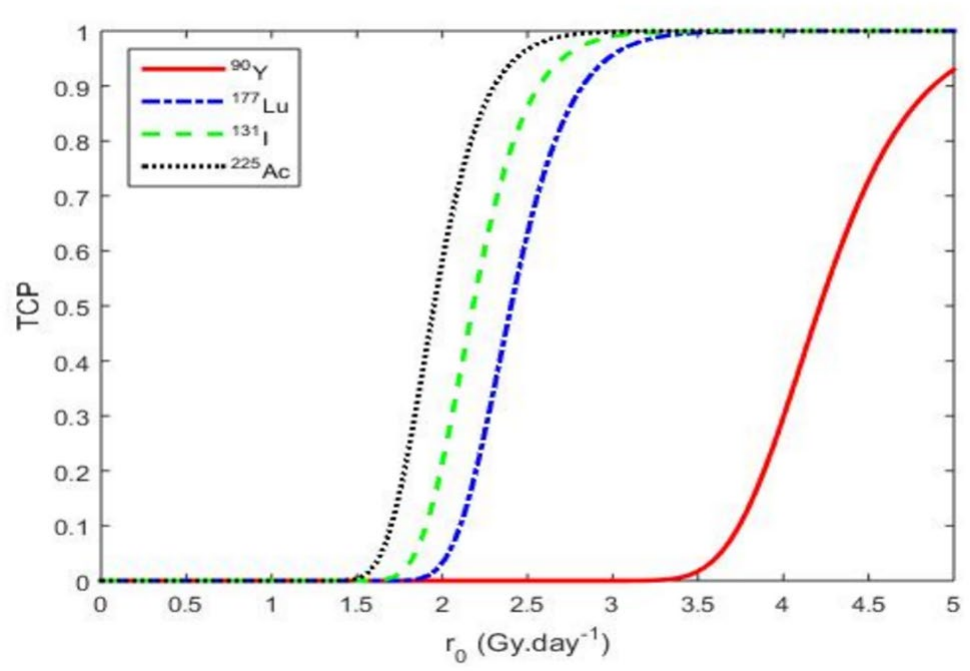
Evolution of TCP as a function of the initial extrapolated dose rate $r_{0}$ for a tumor volume of 100000 $\hbox {mm}^{3}$ and a CSC concentration *S* = 0.004576 cell.$\hbox {mm}^{-3}$. The values of the other parameters are given in Table 4.

From Eq. (11) without the $\mathrm {OER(P_{0})}$, we plot the TCP curve in Fig. 5. It shows that among the four radionuclides the one with the longest half-life achieves a TCP of 1 at the lowest dose rate for a tumor diameter of 10 mm. This suggests a faster and more effective tumor control potential with this radionuclide. From the clinical point of view, the TCP = 1 indicates a more effective therapy at lower doses with reduced exposure to healthy tissues^84–86^. This can improve patient tolerance and quality of life, particularly in aggressive or resistant tumors. From the Fig. 5, the TCP curve of $^{225}\textrm{Ac}$ (3.485 $\mathrm {Gy.day^{-1}}$) reaches the value 1 before those of $^{90}\textrm{Y}$ (above 5 $\mathrm {Gy.day^{-1}}$), $^{177}\textrm{Lu}$ (4.192 $\mathrm {Gy.day^{-1}}$), and $^{131}\textrm{I}$ (3.838 $\mathrm {Gy.day^{-1}}$)., it indicates greater efficacy at a lower dose (3.485 $\mathrm {Gy.day^{-1}}$). This result suggests that the $^{225}\textrm{Ac}$ can be more appropriate for personalized treatment compared to the other radionuclides used in this work.

### The simulations with oxygen effects

In this section, we study the oxygen effects on CSCs and each radionuclide. For this, we use Eqs. (4), (9) and (11).

**Fig. 6:**
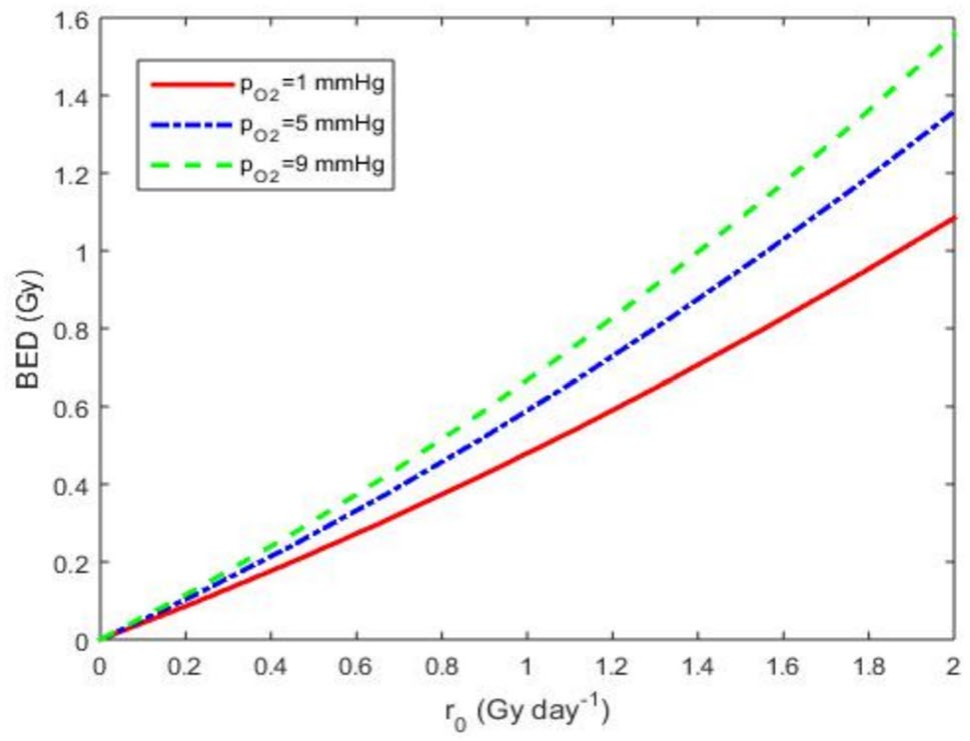
Evolution of BED as a function of the extrapolated initial dose rate $r_{0}$, under varying oxygen pressures. The values of all other parameters are provided in Table 4.

From the second Eq. (9), we plot the BED curve with oxygen effects in Fig. 6 which shows the impact of oxygen pressure on the radiation sensitivity of the radionuclide $^{90}\textrm{Y}$. We observe in this figure that cancer tissues (9 mmHg) are more sensitive to radiation because oxygen enhances the effects of radiation by promoting the formation of free radicals that damage DNA. In contrast, hypoxic cancer tissues (1 mmHg) are more resistant to radiation as the lack of oxygen reduces the formation of free radicals and consequently the DNA damage. At a given dose rate $r _{0}$, the BED is higher for well-oxygenated cancer tissues (9 mmHg) compared to hypoxic cancer tissues (1 mmHg). This indicates that a specific radiation dose rate is more effective (in terms of biological damage) under well-oxygenated conditions. Furthermore, the graphs demonstrate that well-oxygenated tumor areas respond better to RIT than hypoxic areas. Consequently, hypoxic tissues may diminish the effectiveness of RIT.

**Fig. 7:**
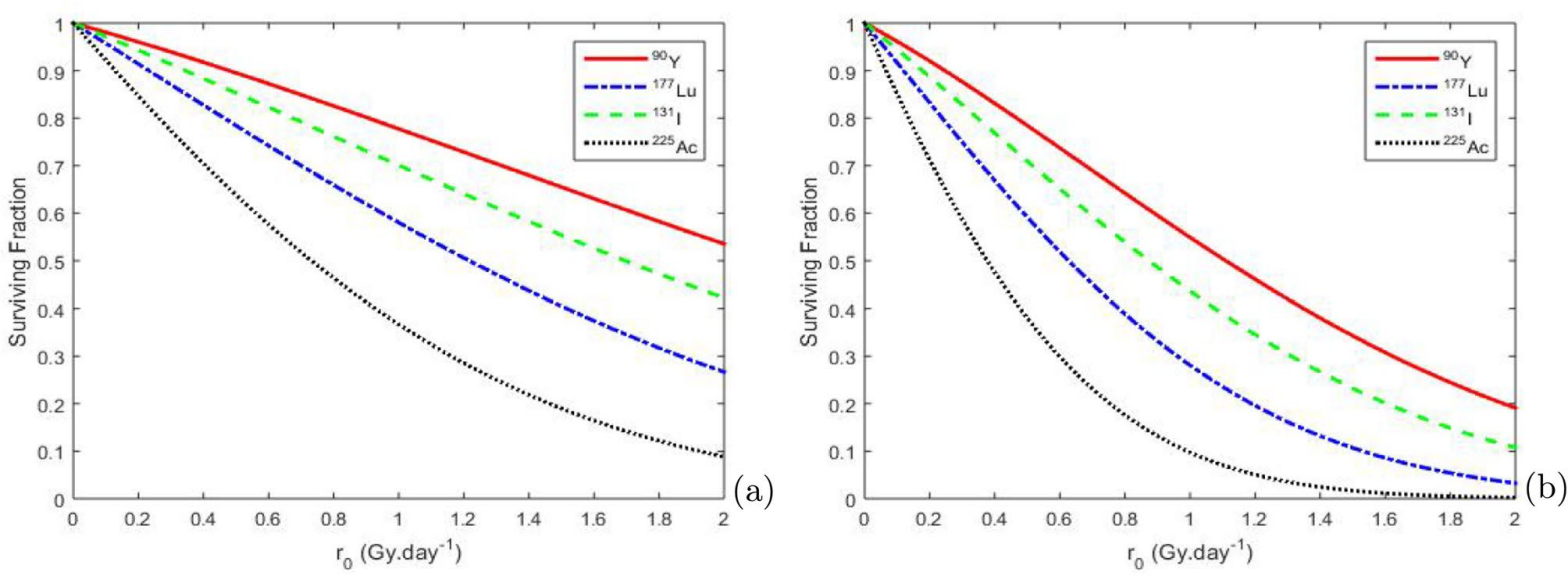
Variation of SF as a function of the extrapolated initial dose rate $r _{0}$ for an $O_{2}$ partial pressure $P_{o}$ = 1 mmHg (**a**) and $P_{o}$ = 40 mmHg (**b**), with the values of the other parameters are given Table 4.

Figure 7 is plotted from Eq. (6). It illustrates the variations in the surviving fraction (SF) of CSCs as a function of the extrapolated initial dose, highlighting the influence of different partial oxygen pressure values in the context of radionuclide exposure. Table 7 summarizes the observed SF values for CSCs under the influence of radionuclides, taking into account the partial pressure of oxygen for a dose $r _{0}$ = 2 $\mathrm {Gy.year^{-1}}$.

**Table 7:** Values of the surviving fraction.

Radionuclide	SF value for $P_{o}$ = 1 mmHg	SF value for $P_{o}$ = 40 mmHg
$^{225}\textrm{Ac}$	0.0886	0.0020
$^{131}\textrm{I}$	0.4217	0.1074
$^{177}\textrm{Lu}$	0.2666	0.0327
$^{90}\textrm{Y}$	0.5354	0.1909

These values indicate that the SF decreases with increasing oxygen levels for each radionuclide, and that $^{225}\textrm{Ac}$ is the most effective.

**Fig. 8:**
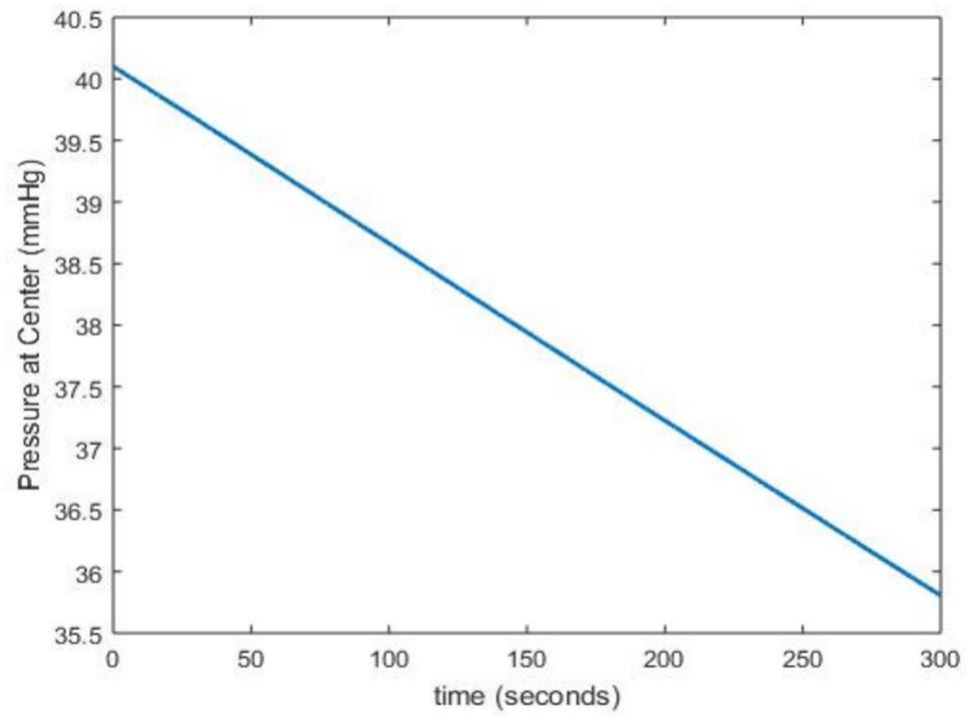
Evolution of oxygen pressure as a function of time for a cell concentration $(S + D )$ = 0.001 $\mathrm {cell.mm^{-3}}$, using the parameter values from Tables 3 and 4.

From Eq. (4), we plot Fig. 8 which shows the decrease in oxygen pressure over time in a tumor. Biomedically, the observed decrease indicates the development of hypoxic regions, which occur when the consumption of oxygen by rapidly proliferating tumor cells exceeds the local oxygen supply from surrounding vasculature. These hypoxic zones can reduce the effectiveness of therapies such as radiotherapy, as oxygen is required to enhance the production of reactive oxygen species that damage tumor DNA^87^. It also promotes the selection of more aggressive cancer clones and potentially metastasis^88,89^. Understanding this dynamic allows for the development of therapeutic strategies to reoxygenate hypoxic areas, thereby improving the effectiveness of treatments and patient outcomes.

**Fig. 9:**
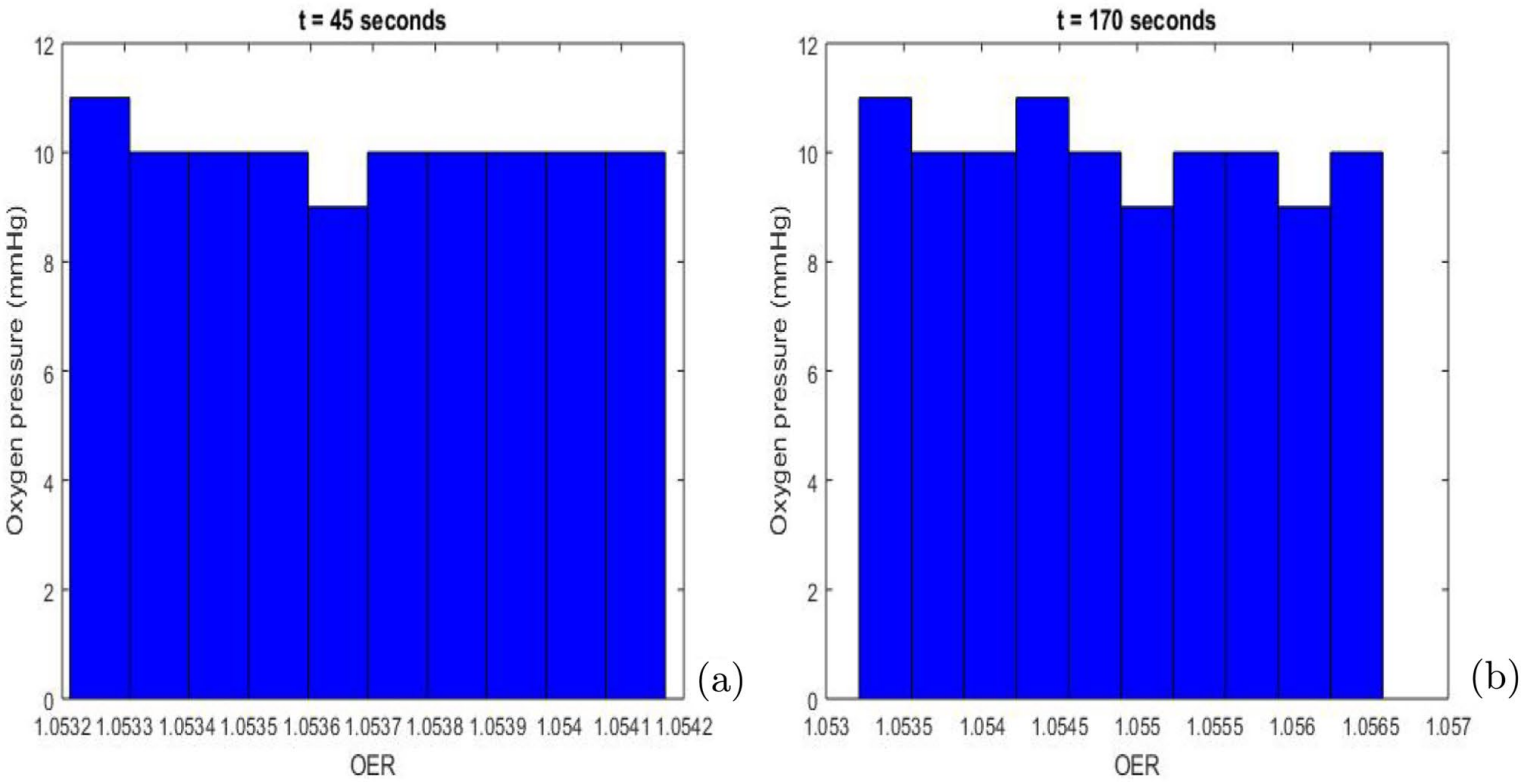
Histogram of oxygen pressure as a function of *OER* at *t* = 45 seconds (Fig. 9 (**a**)) and at *t* = 170 seconds (Fig. 9 (**b**)). $OER_{\text {max}} = 3$ and $K_{m}$ = 3.28 mmHg.

Figure 9 presents the histogram of oxygen pressure as a function of OER at *t* = 45 seconds (Fig. 9 (a)) and at *t* = 170 seconds (Fig. 9 (b)). The data in the histogram allow visualizing the distribution of oxygenation levels and estimating which regions of the tumor are well oxygenated (and thus more radiosensitive) compared to those that are hypoxic (and less radiosensitive). A high oxygen pressure at high OER values indicates that a large proportion of cells in the tumor are well oxygenated, meaning that radiation will potentially be more effective in these regions. Conversely, a low oxygen pressure at high OER values suggests that there are fewer well-oxygenated cells in the tumor, making these regions less sensitive to radiation and therefore more challenging to treat effectively.

On the other hand, a high oxygen pressure at low OER values indicates a prevalence of hypoxic cells in the tumor. Hypoxic cells are more resistant to radiation, which could indicate a less favorable prognosis for radiation treatment, potentially requiring higher doses or additional treatments.

The distribution of OER provides insights into the potential effectiveness of radiation. Well-oxygenated regions (high OER) are more likely to respond favorably to treatment. Therefore, knowing the distribution of OER helps in planning the radiation dose and considering additional strategies to target hypoxic regions. Moreover, the OER histogram can serve as a guide for evaluating prognosis and adapting therapeutic protocols to improve the chances of treatment success.

**Fig. 10:**
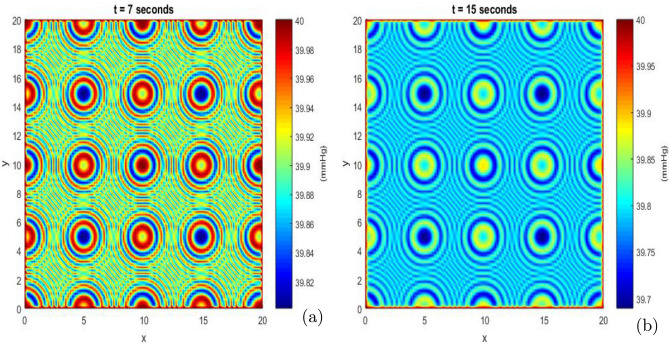
Process of circular patterns in oxygen pressure distribution at *t* = 7 seconds (**a**) and at *t*= 15 seconds (**b**) for a cell concentration $( S + D)$ = 0.001 $\mathrm {cell.mm^{-3}}$. All model parameters are provided in Table 3.

Figures 10 (a) and 10 (b) show the formation of circular patterns in the distribution of oxygen pressure observed in solid cancers. These circular patterns emerge around hypoxic zones and reflect the heterogeneity of oxygenation within the tumor microenvironment^59,90^. In these hypoxic areas, the partial pressure of oxygen is significantly reduced compared to surrounding regions, contributing to spatially structured low-oxygen niches^91^. Circular patterns of oxygen pressure distribution have been observed in various types of cancers, including solid tumors such as breast cancer, brain cancer, prostate cancer and lung cancer. These patterns can result from complex biological factors such as tumor growth, oxygen consumption and interaction with the tumor microenvironment. These circular patterns are heterogeneous, indicating substantial variations in oxygen levels within the tumor^92^.

The presence of circular patterns in the distribution of partial oxygen pressure $(P_{O})$ within a tumor has significant clinical, biomedical, and biological implications. These patterns can provide insights into tumor behavior, treatment resistance, and the underlying physical processes within the tumor microenvironment.

Clinically, circular patterns of low oxygen often indicate regions of hypoxia (areas with low oxygen levels) within the tumor. This is because oxygen enhances the production of reactive oxygen species (ROS) during radiation therapy, which helps in damaging the DNA of cancer cells. In hypoxic regions, this effect is reduced, leading to lower effectiveness of radiation treatment^91^. The identification of these patterns using imaging techniques (such as PET scans with hypoxia-sensitive tracers) allows for more targeted therapies, including hypoxia-targeted drugs or adjusted radiotherapy protocols to increase radiation doses specifically in hypoxic areas^93^.

Hypoxia is generally associated with a more aggressive tumor phenotype, as it can promote metastatic potential and resistance to chemotherapy. The extent and distribution of these hypoxic zones can therefore serve as a prognostic indicator, helping clinicians assess the likely aggressiveness of the tumor and tailor treatment plans accordingly^45^.

Biomedically, these circular patterns reflect oxygen diffusion-consumption dynamics. Oxygen diffuses from regions of higher concentration to hypoxic zones, creating gradients where oxygen levels are higher near more oxygenated areas and lower in distant regions. This radial diffusion contributes to circular zones of decreasing oxygen concentration^94^.

Biologically, hypoxia triggers adaptive responses in tumor cells, primarily through the activation of hypoxia-inducible factors (HIFs). HIFs regulate the expression of genes involved in metabolism, cell survival, and invasion, enabling tumor cells to thrive under low oxygen. Tumor cells in hypoxic regions often switch from oxidative phosphorylation to glycolysis, a less efficient form of energy production that does not rely on oxygen (known as the Warburg effect). This shift supports cell survival but leads to the accumulation of lactate, acidifying the tumor microenvironment, which can further promote invasion and immune evasion^95^.

The circular patterns of oxygenation reflect the highly heterogeneous nature of the tumor microenvironment, which contains regions with vastly different conditions (for example, hypoxic cores surrounded by better-oxygenated rims). This heterogeneity can drive the evolution of diverse cell populations within the tumor, contributing to its complexity and ability to adapt to therapeutic pressures^45^.

There are moments when oxygenation appears homogeneous in parts of the tumor, depending on factors like local cell density and tissue architecture^96^. Understanding these patterns is essential to optimize therapeutic strategies and improve treatment efficacy while reducing tumor resistance^96,97^.

### The time series profiles of CSCs, DCs, Wnt-$\beta$ catenin and microRNAs in the absence of therapy

Here, we present the time evolution of CSCs, DCs, Wnt-$\beta$ catenin and microRNAs concentration profiles, using Eq. (1) without RIT.

**Fig. 11:**
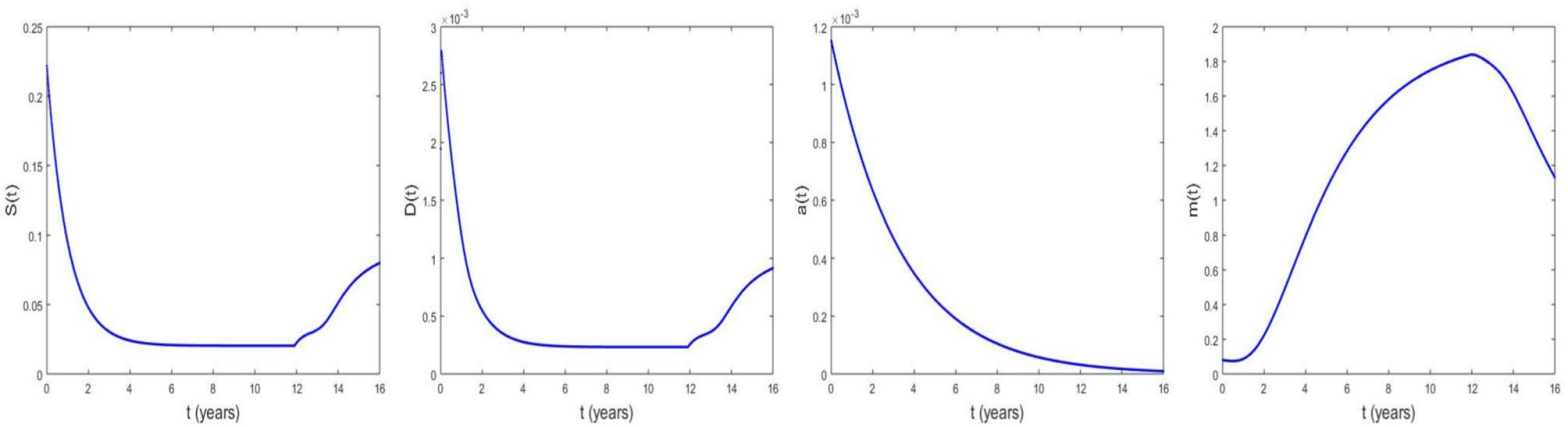
Time series of CSCs, DCs, Wnt-$\beta$ and microRNAs in the abscence of therapy at $\tau _{c}$ = 11.8964 years for $t =$ 16 years and the values of other parameters are given in Table 2.

Figure 11 shows that at recurrence period $\tau _{c}$ = 11.8964 *years* for prostate cancer, the oncogenic behavior of the microRNAs emerges, resulting in a decrease in their concentration, which leads to an increase in the concentrations of CSCs and DCs. In contrast, the Wnt-$\beta$ catenin pathway remains inactive as indicated by the lack of growth in its concentration.

### The radioimmunotherapy effects on CSCs, DCs, Wnt-$\beta$ catenin and microRNAs

In this section, we describe the RIT effects on the dynamical system using Eq. (1).

**Fig. 12:**
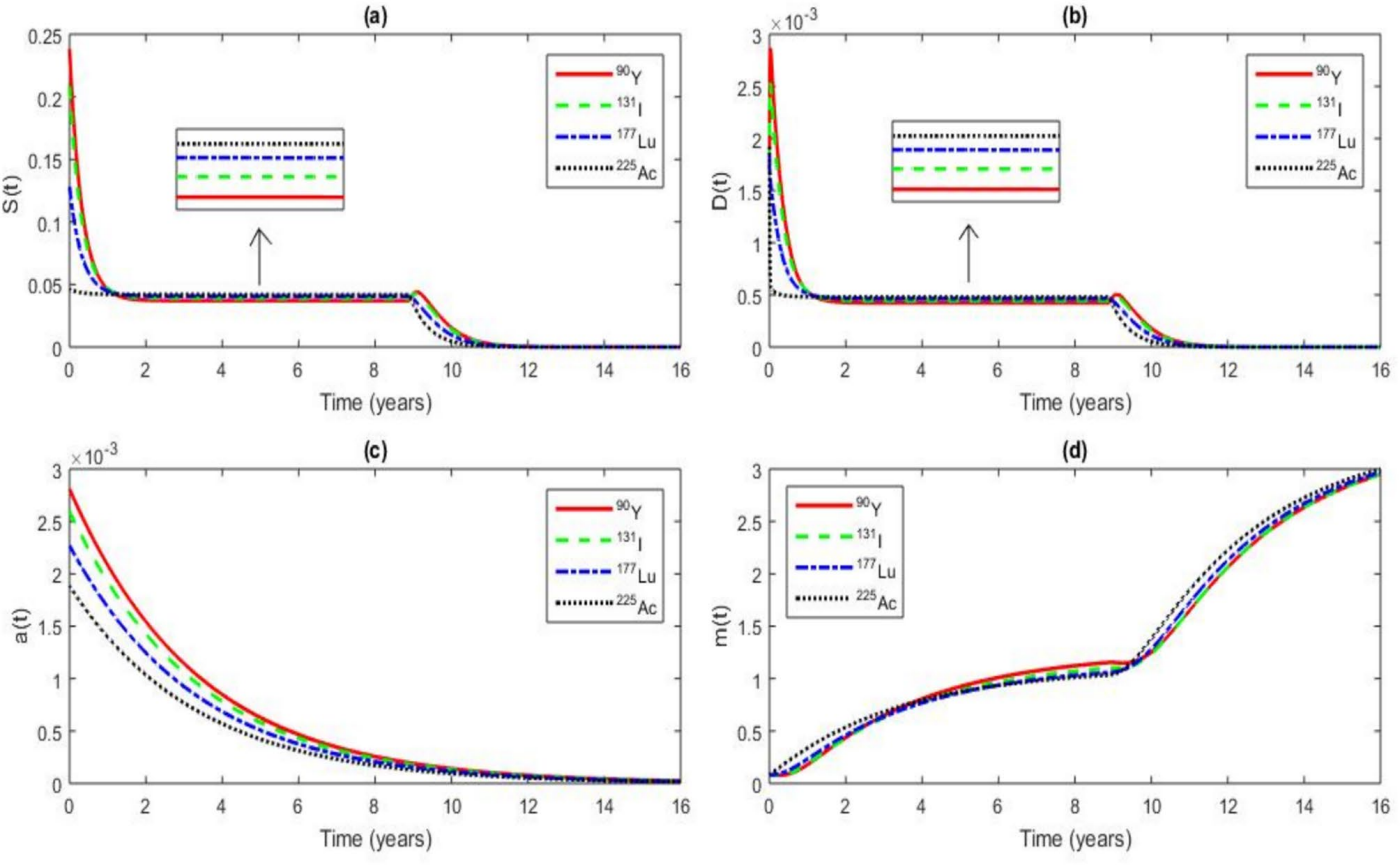
RIT effects on the time series of *S*(*t*), *D*(*t*), *a*(*t*) and *m*(*t*) for a value of $\tau$ = 8.8964 years $< \tau _{c}$ = 11.8964 years at $t = 16$ years, $r_{0}$ = 20 $\mathrm {Gy.year^{-1}}$, with the other parameter values given in Tables 2 and 3. The analysis focuses on the radionuclides $^{90}\textrm{Y}$, $^{131}\textrm{I}$, $^{177}\textrm{Lu}$ and $^{225}\textrm{Ac}$.

Figure 12 illustrates the effects of RIT on CSCs and DCs, using an initial dose of $r_0$ = 20 $\mathrm {Gy.year^{-1}}$. RIT inhibits the conversion of DCs into CSCs, a critical mechanism for CSC eradication, and also influences the Wnt-$\beta$ catenin pathway and microRNA expression. However, this figure highlights a transient CSC and DC repopulation phase during RIT, occurring over 40 days (from $t = 8.8964$ years to $t = 9.006$ years). This phenomenon has been previously observed in prostate cancer^98^ and is directly induced by RIT. Unlike external beam radiotherapy, which delivers radiation in discrete fractions, RIT provides continuous irradiation over an extended period. As a result, CSCs and DCs are not immediately eradicated but instead undergo an initial stimulation phase. This transient stimulation arises from the radiation-induced activation of cellular survival mechanisms, such as DNA damage response pathways (ATM, ATR, p53) and the Wnt-$\beta$ catenin signaling pathway, which are critical for CSC maintenance. Additionally, low-dose radiation has been shown to induce compensatory proliferation in tumor cells as part of a repopulation response^99,100^. This effect is particularly pronounced in radiotherapy when tumor cells experience sublethal doses, leading to increased proliferation before their eventual decline^101^. In the case of RIT, this repopulation effect is more gradual due to the continuous exposure to radionuclides, explaining the transient increase in CSC and DC concentrations before their eventual reduction. Initially, RIT stimulates both CSCs and DCs, but their populations decline over time, accompanied by a decrease in microRNA levels. Table 8 provides quantitative data on CSC, DC, and microRNA concentrations at $t = 9.006$ years.

**Table 8:** Values of the CSC, DC and microRNA concentrations at $t = 9.006$ years when $r_{0}$ = 20 $\mathrm {Gy.year^{-1}}$.

Radionuclide	Concentration value of CSCs ($\mathrm {cell.mm^{-3}}$)	Concentration value of DCs ($\mathrm {cell.mm^{-3}}$)	Concentration value of microRNAs ($\mathrm {molecule.mm^{-3}}$)
$^{225}\textrm{Ac}$	0.0338857	0.0003830	1.0407905
$^{131}\textrm{I}$	0.0419344	0.0004678	1.1021730
$^{177}\textrm{Lu}$	0.0381324	0.0004279	1.0616104
$^{90}\textrm{Y}$	0.0425007	0.0004730	1.1545671

In Fig. 12 (a), at $t = 9.796$ years, $^{225}\textrm{Ac}$ induces a 80.08% reduction in CSCs, compared to 65.15% for $^{177}\textrm{Lu}$, 55.04% for $^{131}\textrm{I}$, and 51.21% for $^{90}\textrm{Y}$ relative to their values at $t = 9.006$ years. These results suggest that among the evaluated radionuclides, $^{225}\textrm{Ac}$ exhibits the most pronounced and rapid CSC eradication effect. Since CSCs play a crucial role in tumor persistence and therapeutic resistance, this finding highlights $^{225}\textrm{Ac}$ as a potent therapeutic agent for highly resistant cancers, including prostate cancer. For instance, $^{225}\textrm{Ac}$-PSMA therapy is currently being investigated in clinical trials for metastatic castration-resistant prostate cancer (mCRPC), showing promising efficacy (NCT06229366, NCT05983198)^102,103^. Additionally, preclinical studies have demonstrated the efficacy of an $^{225}\textrm{Ac}$-labeled anti-CD45 monoclonal antibody in eliminating CD45+ cells in a human multiple myeloma model^104^. Furthermore, Phase I clinical trials have explored $^{225}\textrm{Ac}$-conjugated antibodies targeting the CD33 antigen for acute myeloid leukemia (AML), reporting significant anti-leukemic activity and suggesting its potential for eliminating leukemic stem cells^105^.

Figure 12 (b) shows that at $t = 9.796$ years, all four radionuclides significantly reduce DC concentrations, limiting CSC replenishment and thus decreasing the risk of tumor recurrence. Specifically, $^{225}\textrm{Ac}$ reduces DCs by 80.08%, $^{177}\textrm{Lu}$ by 64.97%, $^{131}\textrm{I}$ by 54.57%, and $^{90}\textrm{Y}$ by 50.61% when $r_0$ = 20 $\mathrm {Gy.year^{-1}}$ compared to their values at $t = 9.006$ years. Targeting the DC to CSC conversion could reduce tumor recurrence risk, as demonstrated in AML treatment with $^{225}\textrm{Ac}$-conjugated antibodies^105^.

Figure 12 (c) shows that all radionuclides inhibit the Wnt-$\beta$ catenin pathway, which is crucial for CSC survival. $^{225}\textrm{Ac}$ has the strongest effect, highlighting its potential for treating resistant cancers like prostate cancer.

Figure 12 (d) shows that RIT significantly increases tumor-suppressive microRNAs after the repopulation. This upregulation, linked to a decrease in both CSC and DC populations, enhances the effectiveness of radiotherapy. For example, miR-34a and miR-200, known regulators of CSC survival and differentiation, show increased expression levels^30^, which correlate with a reduction in both CSC and DC populations. The pronounced miRNA response observed with $^{225}\textrm{Ac}$ suggests that this radionuclide exerts a dual cytotoxic effect: direct radiation-induced damage and indirect suppression of molecular pathways supporting CSCs.

Collectively, these findings highlight the potential of radionuclide-based therapy to target key mechanisms of tumor resistance and recurrence. By effectively reducing CSC populations, suppressing Wnt-$\beta$ catenin signaling, and modulating microRNA expression, radionuclides such as $^{225}\textrm{Ac}$ not only enhance the direct cytotoxic effects of radiation but also provide a means to disrupt the molecular pathways that sustain CSC-driven tumor progression. The following summary in Table 9 shows the CSC, DC and microRNA concentration values for each radionuclide.

**Table 9:** Values of the CSC, DC and microRNA concentrations at $t = 9.796$ years when $r_{0}$ = 20 $\mathrm {Gy.year ^{-1}}$.

Radionuclide	Concentration value of CSCs ($\mathrm {cell.mm^{-3}}$)	Concentration value of DCs ($\mathrm {cell.mm^{-3}}$)	Concentration value of microRNAs ($\mathrm {molecule.mm^{-3}}$)
$^{225}\textrm{Ac}$	0.0067512	0.0000763	1.2875087
$^{131}\textrm{I}$	0.0188551	0.0002125	1.1841774
$^{177}\textrm{Lu}$	0.0132884	0.0001499	1.2143314
$^{90}\textrm{Y}$	0.0207363	0.0002336	1.2076104

The observed superiority of $^{225}\textrm{Ac}$ in eradicating CSCs highlights its potential as a targeted therapy for tumors that exhibit intrinsic resistance to conventional radiotherapy^106,107^. The marked increase in tumor-suppressive microRNAs suggests that radionuclide therapy could be combined with microRNA-based therapeutic strategies to further enhance tumor control (National Cancer Institute). The sustained suppression of the Wnt pathway implies that these radionuclides could sensitize CSCs to other therapeutic modalities, such as small molecule inhibitors targeting CSC-associated pathways^108^. Furthermore, the differential effects of $^{225}\textrm{Ac}$, $^{177}\textrm{Lu}$,$^{131}\textrm{I}$, and $^{90}\textrm{Y}$ on CSC depletion, DC suppression, and microRNA modulation suggest that patient-specific radionuclide selection could optimize therapeutic outcomes.

**Fig. 13:**
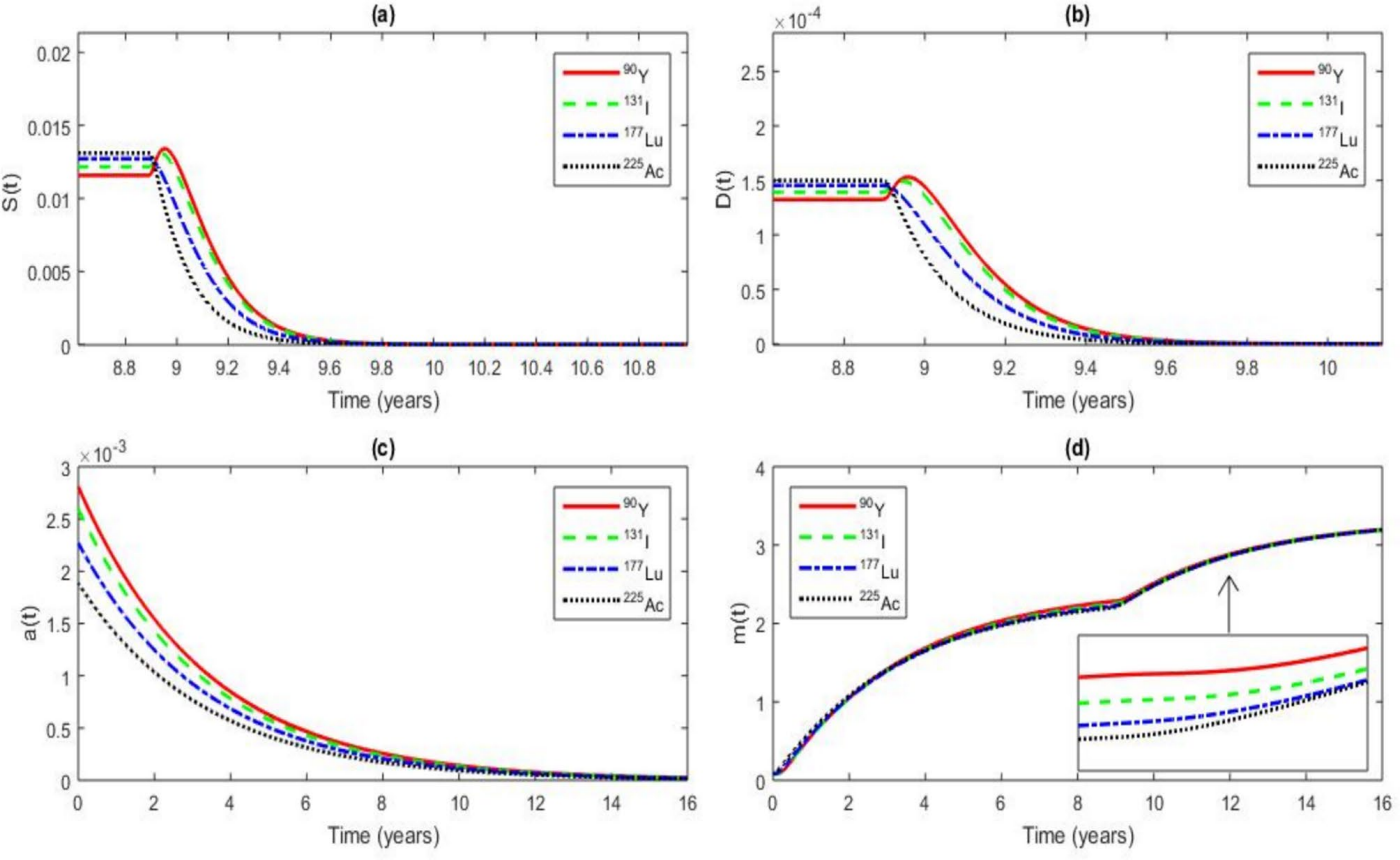
RIT effects on the time series of *S*(*t*), *D*(*t*), *a*(*t*) and *m*(*t*) for a value of $\tau$ = 8.8964 years $< \tau _{c}$ = 11.8964 years at $t = 16$ years, $r_{0}$ = 165 $\mathrm {Gy.year^{-1} }$. The parameter values are given in Tables 1 and 2. The analysis focuses on the radionuclides $^{90}\textrm{Y}$, $^{131}\textrm{I}$, $^{177}\textrm{Lu}$ and $^{225}\textrm{Ac}$.

Figure 13 illustrates the impact of RIT on CSCs, DCs, the Wnt-$\beta$ catenin signaling pathway, and microRNA expression, considering an extrapolated initial dose of $r_{0}$ = 165 $\mathrm {Gy.year^{-1}}$.

Figure 13 (a) shows that for $r_{0}$ = 165 $\mathrm {Gy.year^{-1}}$, CSC burden significantly decreases at $t = 9.796$ years for all radionuclides compared to the values obtained at the same time point with $r_{0}$ = 165 $\mathrm {Gy.year^{-1}}$. The corresponding CSC concentrations are summarized in Table 10. Moreover, complete and rapid CSC eradication is observed at $t = 10.36$ years for $^{225}\textrm{Ac}$ and $t = 10.47$ years for $^{177}\textrm{Lu}$, indicating superior efficacy of these radionuclides in targeting tumor-initiating cells.

Figure 13 (b) highlights a substantial reduction in DCs under $r_{0}=$ 165 $\mathrm {Gy.year^{-1}}$, reinforcing the hypothesis that depletion of differentiated tumor cells limits CSC repopulation potential. The corresponding DC concentrations at $t=9.796$ years are provided in Table 10.

Figure 13 (c) reveals that the Wnt-$\beta$ catenin pathway remains inactive under high-dose RIT exposure ($r_{0}$ = 165 $\mathrm {Gy.year^{-1}}$). This inactivation is characterized by a progressive decline in Wnt-$\beta$ catenin levels, a key driver of CSC maintenance and tumor progression.

Finally, Fig. 13 (d) illustrates the microRNA-mediated tumor suppressive response following RIT. A significant upregulation of microRNAs is observed at $t = 9.796$ years for all radionuclides compared to their levels at $t = 9.796$ years when $r_{0}$ = 20 $\mathrm {Gy.year^{-1}}$. This dynamic response may reflect a cellular adaptation mechanism that contributes to post-transcriptional regulation of CSC proliferation and differentiation. The specific microRNA concentrations at $t = 9.796$ years are summarized in Table 10.

**Table 10:** Values of the CSC, DC and microRNA concentrations at t = 9.796 $\textrm{years}$ when $r_{0}$ = 165 $\mathrm {Gy.year ^{-1}}$.

Radionuclide	Concentration value of CSCs ($\mathrm {cell.mm^{-3}}$)	Concentration value of DCs ($\mathrm {cell.mm^{-3}}$)	Concentration value of microRNAs ($\mathrm {molecule.mm^{-3}}$)
$^{225}\textrm{Ac}$	0.00001072	0.00000012	2.43122135
$^{131}\textrm{I}$	0.00003968	0.00000043	2.44240734
$^{177}\textrm{Lu}$	0.00002559	0.00000028	2.43175515
$^{90}\textrm{Y}$	0.00004486	0.00000049	2.46482185

From Figs. [12 (a) and 12 (b)] and [Figs. 13 (a) and 13 (b)], we observe that the concentrations of CSCs and DCs have a similar decreasing profile as time progresses. These profiles should be similar if the same initial concentrations of both CSCs and DCs are used. Then, from the biological point of view, CSCs and DCs can be killed at the same rate. This result is a good agreement with the one obtained by Jandl T et al.^19^ who showed that CSCs are killed at the same rate as melanoma cells using radioimmunotherapy.

The results also show that the model predictions are consistent with the recent experimental observations reported by Giri et al.^20^, who showed that alpha-emitting radionuclides ($^{225}\textrm{Ac}$) induce more rapid CSC depletion compared to beta-emitters ($^{177}\textrm{Lu}$).

**Fig. 14:**
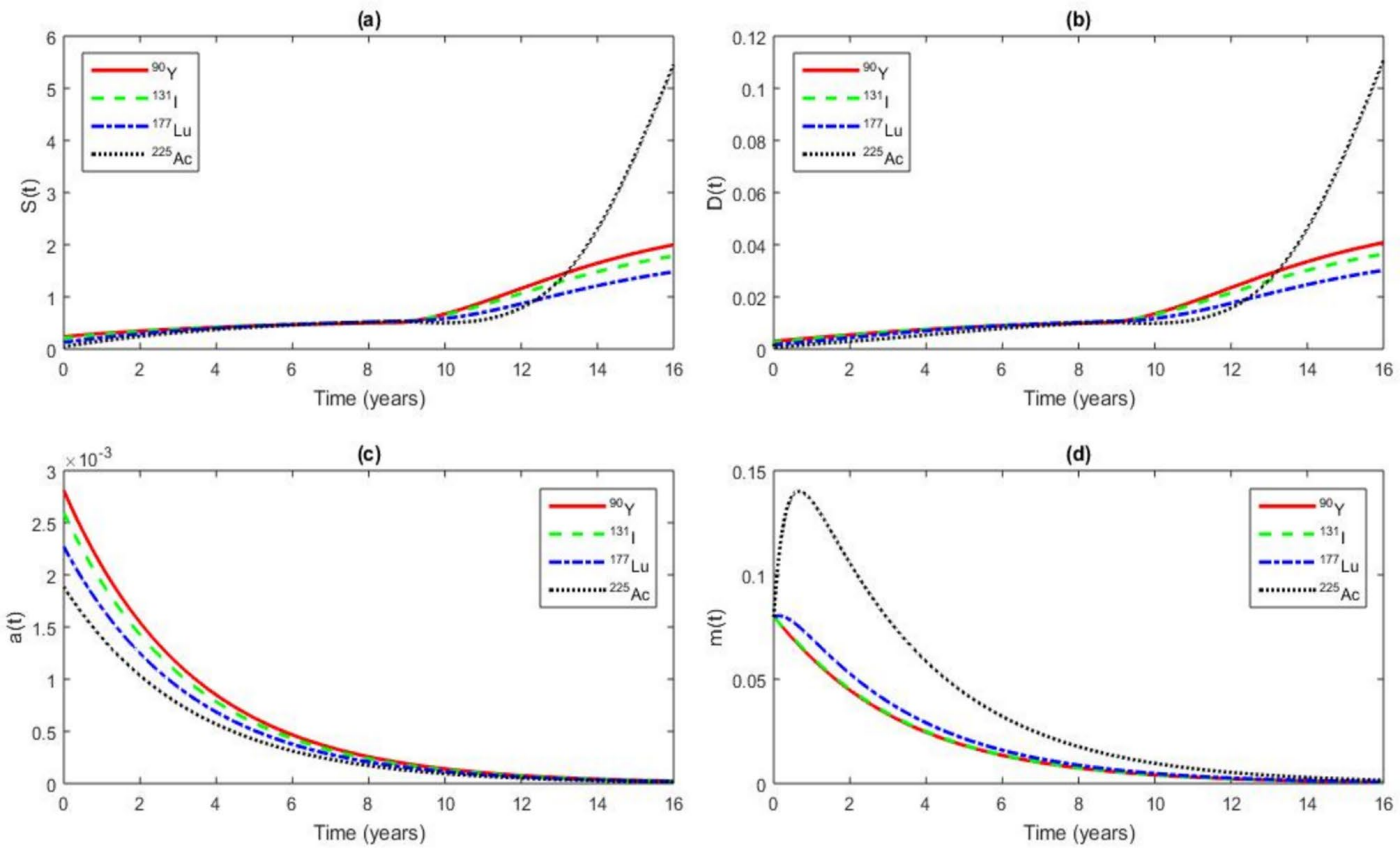
RIT effects on the time series of *S*(*t*), *D*(*t*), *a*(*t*) and *m*(*t*) for a value of $\tau$ = 8.8964 years $< \tau _{c}$ = 11.8964 years at $t = 16$ years, $r_{0}$ = 326 $\mathrm {Gy.year^{-1}}$, the other parameter values are given in Tables 1 and 2. The analysis focuses on the radionuclides $^{90}\textrm{Y}$, $^{131}\textrm{I}$, $^{177}\textrm{Lu}$ and $^{225}\textrm{Ac}$.

Figure 14 shows when the extrapolated initial dose rate is excessively high (for example 326 $\mathrm {Gy.year^{-1}}$), it can still provoke excessive CSCs proliferation. This occurs because extremely high doses can induce stress responses in CSCs and microRNAs leading to an oncogenic behavior that promotes rapid tumor growth and resistance to treatment. For example, Fig. 14 (a) shows that, based on an extrapolated initial dose value of $r_{0}$ = 326 $\mathrm {Gy.year^{-1}}$, excessive proliferation of CSCs occurs following the administration of RIT at $t = 8.8964$ *years* which corresponds to 2 years prior to cancer recurrence. Notably, this proliferation is markedly enhanced by $^{90}\textrm{Y}$ and $^{131}\textrm{I}$ before cancer recurrence time *t* = $\tau _{c}$ = 11.8964 *years*. In contrast, this proliferation is less rapid with $^{225}\textrm{Ac}$ and $^{177}\textrm{Lu}$ before cancer recurrence time *t* = $\tau _{c}$ = 11.8964 years. Figure 14 (b) illustrates that, based on the extrapolated initial dose value $r_{0}$ = 326 $\mathrm {Gy.year^{-1}}$, all DCs convert into CSCs more rapidly with $^{90}\textrm{Y}$ and $^{131}\textrm{I}$, while $^{225}\textrm{Ac}$ and $^{177}\textrm{Lu}$ induce this conversion at a slower rate before cancer recurrence time *t* = $\tau _{c}$ = 11.8964 years. Figure 14 (c) illustrates the absence of activation of the Wnt-$\beta$ catenin pathway, as indicated by the lack of increase in concentration of *a*, despite a very high extrapolated initial dose for radionuclides $^{90}\textrm{Y}$, $^{131}\textrm{I}$, $^{177}\textrm{Lu}$ and $^{225}\textrm{Ac}$. This non-activation is significant, as it helps prevent a highly aggressive and uncontrolled proliferation of CSCs^109^. Consequently, this could lead to a reduction in the aggressive nature of tumor. In Fig. 14 (d), microRNAs act as oncogenes, leading to a decrease in the concentration of *m* from the extrapolated initial dose of 326 $\mathrm {Gy.year ^{-1}}$ for radionuclides $^{90}\textrm{Y}$, $^{131}\textrm{I}$, $^{177}\textrm{Lu}$ and $^{225}\textrm{Ac}$. At this dose, microRNAs exhibit a stronger oncogenic effect with $^{177}\textrm{Lu}$ and $^{225}\textrm{Ac}$ compared to $^{90}\textrm{Y}$, $^{131}\textrm{I}$. This oncogenic behavior promotes CSCs growth, survival and migration contributing to tumor progression and treatment resistance, making tumors more aggressive and increasing the risk of metastasis.

## Conclusion

In this study, we proposed a framework that integrates mathematical modeling, analytical insights and computational simulations to investigate the therapeutic potential of RIT in targeting CSCs and DCs, with a focus on four clinically relevant radionuclides: $^{90}\textrm{Y}$, $^{177}\textrm{Lu}$, $^{131}\textrm{I}$, and $^{225}\textrm{Ac}$. Our results demonstrate that extrapolated dose rates around 165 $Gy.year^{-1}$ are optimal for achieving complete eradication of CSC and DC with radionuclides $^{225}\textrm{Ac}$ and $^{177}\textrm{Lu}$, while higher doses reduce therapeutic efficacy and trigger undesirable oncogenic reprogramming of microRNAs. Under these optimized conditions, Wnt signaling is actively suppressed, and microRNAs act as tumor suppressors, contributing to long-term tumor control. We further show that oxygen availability significantly enhances CSC radiosensitivity, while hypoxia impairs treatment response, emphasizing the importance of accounting for the tumor microenvironment in therapeutic design.

These findings underline the mechanistic importance of integrating radiobiological dose thresholds, microRNA feedback, and tumor oxygenation into the optimization of RIT. Moreover, they support the potential of using BED, TCP, and spatial modeling to predict treatment outcomes more precisely. A sensitivity analysis was also performed to assess the influence of important biological and biophysical parameters on treatment efficacy. This study reinforces the idea that targeting CSCs is crucial for preventing recurrence and managing tumor heterogeneity, particularly when using radionuclides with favorable decay characteristics and biological half-lives.

However, the current model is subject to some limitations. It is based on generic parameter values extracted from the literature, which may not reflect the full spectrum of inter-patient variability. For example, the radiosensitivity of CSCs or their proliferation rates can differ significantly between tumor types or individuals. Furthermore, the oxygenation model relies solely on diffusion and does not consider vascular heterogeneity, nutrient gradients, or acidic extracellular pH. For example, poorly vascularized regions within a tumor may create hypoxic zones that reduce the effectiveness of radiation, particularly for short-range $\alpha$ -emitters like $^{225}\textrm{Ac}$. Finally, the absence of experimental validation limits the immediate clinical applicability of the findings. For future work, we will integrate preclinical data and patient-specific biomarkers to refine and validate the model.

In conclusion, this study supports for the use of $^{225}\textrm{Ac}$ and $^{177}\textrm{Lu}$ in RIT protocols and highlights key biological and biophysical parameters that govern treatment success or failure. The integration of oxygenation effects, dose thresholds, and cellular plasticity provides a powerful basis for optimizing therapeutic strategies. Ultimately, these findings could support the development of next-generation radiolabeled compounds with improved pharmacokinetics and selective CSC targeting, offering new avenues for personalized and effective cancer treatment.

## Data Availability

All data generated or analyzed during this study are included in this paper.
